# Human Purkinje cells outperform mouse Purkinje cells in dendritic complexity and computational capacity

**DOI:** 10.1038/s42003-023-05689-y

**Published:** 2024-01-02

**Authors:** Stefano Masoli, Diana Sanchez-Ponce, Nora Vrieler, Karin Abu-Haya, Vitaly Lerner, Tal Shahar, Hermina Nedelescu, Martina Francesca Rizza, Ruth Benavides-Piccione, Javier DeFelipe, Yosef Yarom, Alberto Munoz, Egidio D’Angelo

**Affiliations:** 1 Department of Brain and Behavioral Sciences, University of Pavia, Pavia, Italy; 2 Centro de Tecnología Biomédica (CTB), Universidad Politécnica de Madrid, Madrid, Spain; 3 Feinberg school of Medicine, Northwestern University, Chicago, IL USA; 4 Department of Neurobiology and ELSC, Edmond J. Safra Campus, The Hebrew University of Jerusalem, Jerusalem, Israel; 5 Brain and Cognitive Sciences and Center of Visual Science, University of Rochester, Rochester, NY USA; 6 Department of Neurosurgery, Shaare Zedek Medical Center, Jerusalem, Israel; 7 Scripps Research Institute, La Jolla, CA USA; 8 Instituto Cajal (CSIC), Madrid, Spain; 9 Departamento de Biología Celular, Universidad Complutense de Madrid, Madrid, Spain; 10 Digital Neuroscience Center, IRCCS Mondino Foundation, Pavia, Italy

**Keywords:** Biophysical models, Neurophysiology

## Abstract

Purkinje cells in the cerebellum are among the largest neurons in the brain and have been extensively investigated in rodents. However, their morphological and physiological properties remain poorly understood in humans. In this study, we utilized high-resolution morphological reconstructions and unique electrophysiological recordings of human Purkinje cells ex vivo to generate computational models and estimate computational capacity. An inter-species comparison showed that human Purkinje cell had similar fractal structures but were larger than those of mouse Purkinje cells. Consequently, given a similar spine density (2/μm), human Purkinje cell hosted approximately 7.5 times more dendritic spines than those of mice. Moreover, human Purkinje cells had a higher dendritic complexity than mouse Purkinje cells and usually emitted 2–3 main dendritic trunks instead of one. Intrinsic electro-responsiveness was similar between the two species, but model simulations revealed that the dendrites could process ~6.5 times (n = 51 vs. n = 8) more input patterns in human Purkinje cells than in mouse Purkinje cells. Thus, while human Purkinje cells maintained spike discharge properties similar to those of rodents during evolution, they developed more complex dendrites, enhancing computational capacity.

## Abstract (web summary)

High-resolution morphological reconstructions and computational models of cerebellar Purkinje cells (PCs) from humans and mice suggest that, while PCs in both species maintain similar spike discharge properties, human PCs developed more complex dendrites.

## Introduction

Current knowledge of neuronal functions still relies almost entirely on rodents^1–3^, in which sophisticated electrophysiological and imaging recordings ex vivo are routinely used to determine membrane potential changes, dendritic processing, synaptic transmission, and long-term synaptic plasticity. Among the few studies addressing the functional properties of human neurons, those on pyramidal cells have shown higher dendritic compartmentalization, faster dendritic integration, and stronger dendritic amplification in humans than in rodents^4–7^. However, no studies have yet investigated the neurons of the human cerebellar cortex.

PCs were first described by Johan Evangelista Purkinje in 1837^8^ and then by Camillo Golgi in 1882 and Santiago Ramón y Cajal, who in 1888 discovered that PC dendrites have dendritic spines^9^. The PC is among the largest and most complex neurons of the brain and shows typical morphological and electrophysiological properties^10–22^. In rodents, PCs express a rich complement of ionic channels, synaptic receptors, and intracellular transduction mechanisms that are differentially distributed across neuron subdivisions. The dendritic tree is almost planar and exhibits a series of ramifications, collecting synaptic inputs and conveying currents to the action potential (AP) initiating site in the axonal initial segment (AIS). Rodent PC dendrites are reported to receive 10^4^ excitatory synapses from parallel fibers (pf) and 10^3^ inhibitory synapses from stellate cells (SC)^23,24^, and are thought to operate as perceptrons^25^ through a process of linear encoding^26^. In rodents, advanced computational models have been constructed and used to simulate neuronal responses both in isolation^25,27–36^ and inside microcircuit reconstructions^24,37^. Little is known about human PCs, which appear larger than rodent PCs^38^. However, electrophysiological recordings are not yet available. Thus, we must answer how human PCs discharge and how their structure impacts computational capacity, that is, the ability to combine multiple input patterns to generate specific responses at the output^39–41^.

Because human datasets are far from complete, computational models can be used to fill the gaps caused by missing knowledge and propose specific functional hypotheses^42^. Detailed neuron models can be reconstructed from digital morphologies to generate morpho-electrical equivalents^43,44^, which can subsequently be endowed with cell-specific ionic conductances^45^. Simulations of responses to current injection can then be optimized against electrophysiological recording templates to extract missing information about model-free parameters, such as maximum ionic conductances^46^. In this study, we utilized high-resolution morphological reconstruction of human PCs and unique electrophysiological recordings obtained in acute cerebellar slices from post-surgical cerebellar specimens to generate detailed biophysical models. This allowed us to simulate the electrophysiological response of human PCs under conditions that would otherwise be impractical for the experimental assessment and evaluation of their dendritic complexity and computational capacity.

## Methods

### Human patch clamp recordings

**Fig. 1:**
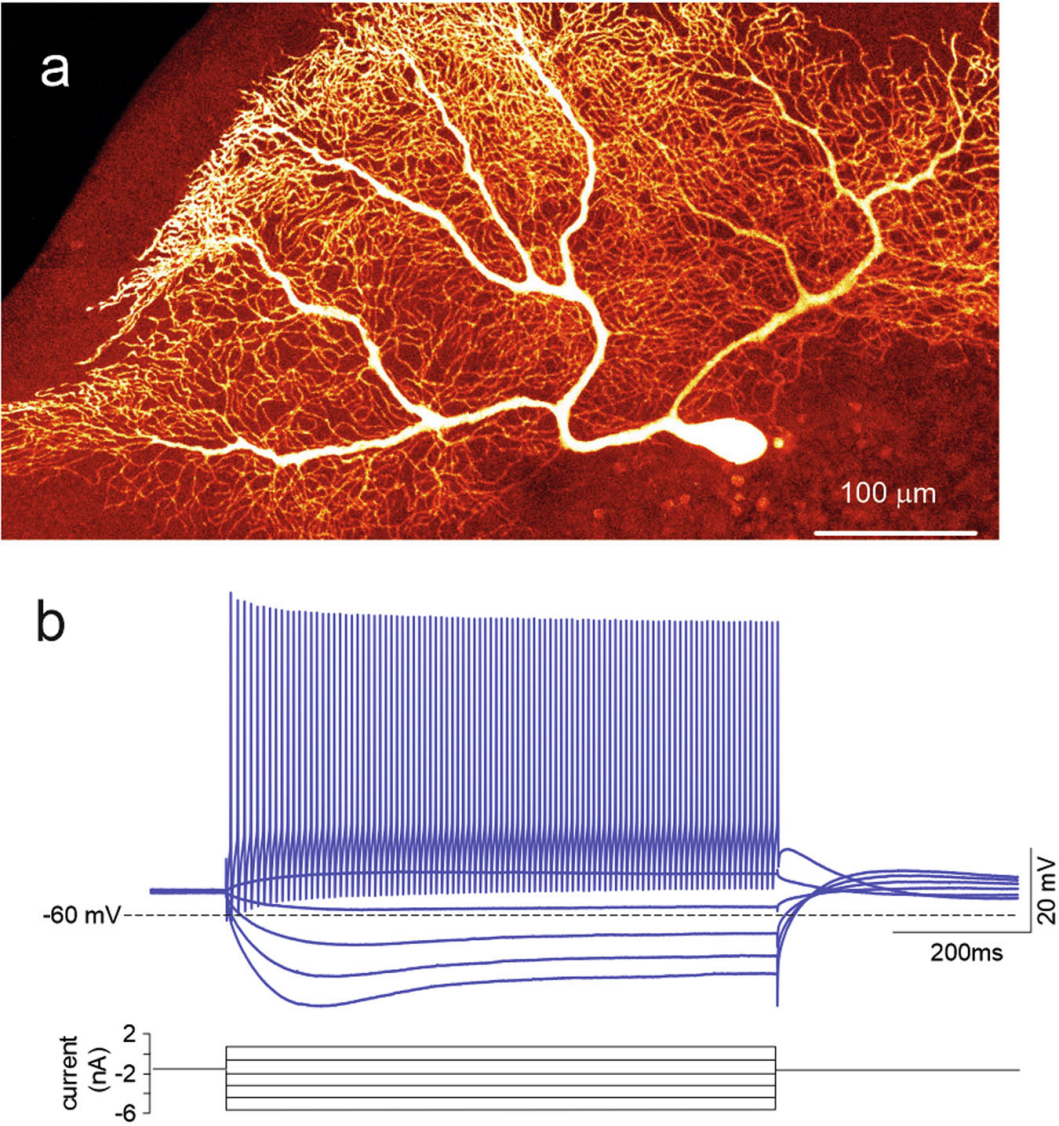
Human PC experimental recordings. **a** Biocytin/streptavidin fluorescence confocal image of a human PC dye-filled with Alexa-647 during a whole-cell patch-clamp recording in an acute cerebellar slice ex vivo. Note the characteristic palisade formed by the dendritic tree that stems with multiple trunks from the soma. The dendritic tree extends for almost 1 millimeter on the parasagittal plane. **b** Example of voltage responses to the injection of positive and negative current steps in the same cell shown in a. Note regular firing in the depolarizing direction and sagging inward rectification in the hyperpolarizing direction.

Ex vivo human cerebellar cortical tissues were obtained from surgeries aimed at deeper brain structures following the guidelines of the institutional ethics committees and the Helsinki Committee of Shaare Zedek Hospital, which also approved the study. The patients signed a consent form to allow the use of a part of their biopsies for scientific research purposes. The tissue samples were considered neurologically normal, as confirmed by a pathologist present during the surgery. Following excision from the brain, cerebellar cortical tissue was placed in ice-cold oxygenated ACSF composed of (in mM) NaCl 126, KCl 2.5, MgSO_4_ 1.5, KH2PO_4_ 1, NaHCO_3_ 24, glucose 10, and CaCl_2_ 1, and bubbled with carbogen (95%O_2_/5%CO_2_) to maintain oxygenation and pH. Following transportation to the laboratory, the pia was removed from the cortical surface as much as possible (without tearing the tissue apart) and tissue chunks were trimmed and/or cut where necessary to expose as much as possible of the translobular plane, while the tissue was maintained submerged in ice-cold oxygenated ACSF. The tissue was then briefly dried and fixed to the stage of a Leica VT1200S vibratome using superglue, along with a piece of agar for structural support. The tissue was then quickly submerged again in ice-cold ACSF continuously bubbled with carbogen, and slices were cut at 300–400 μm thickness at a speed of 0.01–0.04 mm/s and amplitude of 1.25 mm. Slices were incubated for at least 1 h at room temperature in the same solution and then placed in a warmed (~32 °C) recording chamber superfused with oxygenated recording ACSF (same solution as for slicing except with 2.4 mM CaCl_2_). Slices were visualized under an infrared light in an upright microscope with 40x water-immersion objective, and whole-cell patch-clamp recordings of PCs were established using electrodes with 2–6 MΩ resistance pulled from borosilicate glass and filled with an intracellular solution composed of (in mM) K-gluconate 140, HEPES 10, EGTA 0.01, CaCl2 0.001, MgATP 4, and 1% biocytin (w/v), pH was adjusted to 7.2–7.3 using KOH and osmolarity to 290–310 mOsm. Electrical activity was recorded in current-clamp mode at a sampling rate of 20–50 kHz and low-pass filtered at 10 kHz using a MultiClamp 700 B amplifier and Digidata 1550 B digitizer connected to the pClamp software, and stored for offline analysis. Images of the recorded neuronal morphologies were obtained by staining for biocytin using streptavidin conjugated to Alexa-647 and imaging the mounted samples under 10x magnification (see Fig. 1).

### Human morphological reconstructions

Human brain tissue from two autopsy cases (AB6, a 92-year-old female who died from heart failure, and AB7, a 66-year-old male who died from bladder carcinoma) was collected from the Unidad Asociada Neuromax—Laboratorio de Neuroanatomía Humana, Facultad de Medicina, Universidad de Castilla-La Mancha, Albacete (Spain). Brain samples were obtained following the guidelines of the institutional ethics committee, which also approved this study. Cerebellar tissue was considered normal because AB6 and AB7 individuals were free of neurological and psychiatric diseases, although abundant AT8-positive cells were found in the cerebral cortex of the AB6 case^47^. The postmortem delays between death and tissue processing were < 4 h (AB6) and 2.7 h (AB7). Upon removal, the brains were immediately fixed in cold 4% paraformaldehyde in phosphate buffer (PB 0.1 M, pH 7.4) for 24 h. Small blocks from the cerebellar vermis were cut, from which parasagittal sections (300 μm) were obtained with the aid of a Vibratome (Leica).

For intracellular injections, sections were prelabeled with 4,6-diamidino-2-phenylindole (DAPI; Sigma, St Louis, MO), and a continuous current was used to inject Lucifer yellow (8% in 0.1; Tris buffer, pH 7.4; LY) into individual PCs from the vermis of the anterior and posterior lobes. LY was applied to each injected neuron by a continuous current until the distal tips of their branches fluoresced brightly, indicating that the dendrites were completely filled and ensuring that the fluorescence did not diminish at a distance from the soma. Following the intracellular injections, sections were incubated for 72 h at 4 °C in stock solution (2% bovine serum albumin, 1% Triton X-100, and 5% sucrose in PB) containing rabbit anti-LY antibody (1:400 000; generated at the Cajal Institute, CSIC, Madrid, Spain). The sections were then rinsed in PB and incubated in biotinylated donkey anti-rabbit IgG (1:100; Amersham, Buckinghamshire, United Kingdom). The sections were then rinsed again and incubated with Alexa Fluor 488 streptavidin-conjugated antibody (1:1000; Molecular Probes, Eugene, OR, United States). Finally, sections were washed and mounted using ProLong Gold Antifade Reagent (Invitrogen, Carlsbad, CA, USA). See refs. ^48,49^ for further details on the cell injection methodology.

For cell reconstruction and quantitative analysis, the sections were imaged using a confocal scanning laser attached to a fluorescence microscope (Zeiss, LSM710). Fluorescently labeled profiles at different wavelengths were recorded using separate channels. Stacks of images at high magnification (×63 glycerol; voxel size, 0.057 × 0.057 × 0.14 μm^3^) were acquired to capture dendritic arbors on the basis of LY immunostaining. Since intracellular injections of PCs were made in 300 μm-thick parasagittal sections, the part of the dendritic arbor nearest the surface of the slice from which the cell soma was injected (typically at a depth of ~30–50 μm from the surface) could be partially lost. In our case, most of the dendritic arbor was estimated to be included within the section because we only reconstructed PCs with dendritic trees running parallel to the surface of the parasagittal slice, and the geometry of the dendritic trees of PCs was largely flat and restricted to a parasagittal plane^50^ (see Supplementary Fig. 1). All measurements were corrected for tissue shrinkage (correction factor, 0.81).

Neuronal morphology data points for each PC included in the analysis (*n* = 6) were extracted in 3D using Neurolucida 360 (MicroBrightfield). Briefly, dendrites, axons, and somas in the skeleton definition were described using 3D points, delimiting the different segments that form the cell arbor. These points have an associated diameter that provides information on the varying thicknesses of the dendritic or axonal processes at that particular point, and varies along the length of the processes. The soma was defined using a set of connected points by tracing the contour of the soma in 2D.

**Fig. 2:**
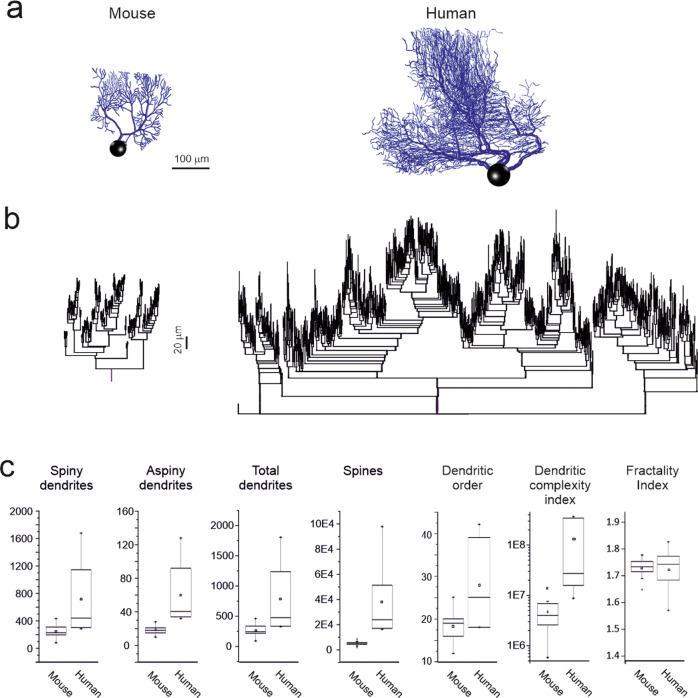
Human and mouse PC morphological properties. **a** Morphological reconstructions of a mouse and a human PC obtained from fluorescent confocal images of postmortem fixated brains (see Supplementary figure 3 for more reconstructions). Note the similar shape but greater size of the human PC compared to the mouse PC. **b** Dendrograms of the two PCs shown above. Note the similar architecture of dendritic ramification. **c** The boxplots show metrics measured from dendrograms of 19 mice and 6 human PCs. These include the number of spiny and aspiny dendrites, the total number of dendrites and spines, and the dendritic order. Note that all these parameters were larger in human than mouse PCs. The dendritic complexity index (DCI) was one order of magnitude higher in human than mouse PCs, while the fractality index was similar in human and mouse PCs. All the comparisons reported in the figure reveal a statistically difference at *p* < 0.01 (unpaired t-test) between human and mouse PC parameters, except for the fractality index. The square at the center of each box define the mean, the line in the box define the median and the x define the Outliers.

To calculate the number and density of spines, spines from all sizes and morphologies were counted in dendritic segments, with diameters decreasing in intervals of 0.1 μm (ranging from 1.2 to 0.2 μm) at different points along the length of the dendrites from five reconstructed PCs. Spine density values in dendrites with different diameters were obtained by dividing the spine count by the length of each segment. These measured density values were used to estimate the total number of spines and the mean density of PCs, taking into consideration the length of the dendritic branches with different diameters over the entire dendritic tree. The dendritic spine structure was analyzed using Imaris 6.4.0 (Bitplane AG, Zurich, Switzerland) with 150 dendritic spines. Because confocal stacks of images intrinsically result in z-dimension distension, a correction factor of 0.84 was applied to that dimension. This factor was calculated using a 4.2 μm Tetraspeck Fluorescent microsphere (Molecular Probes) under the same parameters used for the acquisition of dendritic stacks. Optical deconvolution was not used for spine reconstruction. The spine head area was 3D reconstructed in a selection of spines showing a clear head whose morphology could be captured using a single surface of a particular intensity threshold. The spine neck length and diameter were manually marked in each selected dendritic spine from the point of insertion in the dendritic shaft to the spine head while rotating the image in 3D (see refs. ^49,51^ for further details) (Fig. 2).

### Mouse Patch-clamp recordings

Mouse recordings were performed using P26-P30 C57BL/6 N wild-type mouse cerebellar slices. The mice were anesthetized with halothane (Sigma-Aldrich) and sacrificed by decapitation to remove the cerebellum for acute slice preparation according to a well-established technique. The cerebellar vermis was isolated and fixed to a vibroslicer stage (Leica VT1200S; Leica Biosystems) with cyanoacrylic glue. Acute 220 μm-thick slices were cut in the sagittal plane and, during the slicing procedure, the cerebellar vermis was immersed in a cold (2–3 °C) oxygenated bicarbonate-buffered saline solution (Kreb’s solution) containing (mM): NaCl 120, KCl 2, MgSO_4_ 1.2, NaHCO_3_ 26, KH_2_PO_4_ 1.2, CaCl_2_ 2, glucose 11 (pH 7.4 when equilibrated with 95%O_2_–5%CO_2_). The slices were incubated at room temperature in an oxygenated standard extracellular solution for at least 1 h until use. For whole-cell patch clamp recordings, slices were placed in a chamber continuously perfused at a rate of 1.5 mL/min with oxygenated Kreb’s solution and maintained at 32 °C with a Peltier feedback device (TC-324B, Warner Instrument Corp.). The slices were visualized using an upright epifluorescence microscope (Axioskop 2 FS; Carl Zeiss) equipped with a 63, 0.9 NA water-immersion objective (Olympus, Hamburg, Germany). Whole-cell patch-clamp recordings from the soma of PCs were performed with a Multiclamp 700 B (−3 dB; cutoff frequency [fc], 10 kHz), sampled with a Digidata 1550 interface, and analyzed offline with pClamp10 software (Molecular Devices). Patch pipettes were pulled from borosilicate glass capillaries (Sutter Instruments) and filled with an internal solution containing (in mM) potassium gluconate 126, NaCl 4, HEPES 5, glucose 15, MgSO_4_ 7, H_2_O 1, BAPTA-free 0.1, BAPTA-Ca^2+^ 0.05, Mg^2+^ -ATP 3, Na + -GTP 0.1, pH 7.2, and adjusted with KOH. Pipettes had a resistance of 2–3 MΩ when immersed in the bath. The signals were low-pass filtered at 10 kHz and acquired at 50 kHz.

All procedures were conducted in accordance with the European Guidelines for the Care and Use of Laboratory Animals (Council Directive 2010/63/EU) and approved by the ethics committee of the Italian Ministry of Health (628/2017-PR).

### Mouse morphological reconstructions

The 3D morphologies of 19 PCs were reconstructed from the anterior zone of lobule V of postnatal (P)27 L7-tau-GFP mice using Neurolucida (MBF Bioscience)^19,52^. Age-matched male mice were used to reduce inter-animal anatomical variability (such as folia size). Briefly, following transcardial perfusion (4% paraformaldehyde in 0.1 M phosphate buffer), brains were removed from the skulls, postfixed overnight in the same fixative, and transferred to a 0.01 M phosphate buffer saline (0.9% sodium chloride) solution (PBS). A Leica vibratome was used to collect 80 µm thick parasagittal sections in cold 0.01 M PBS. Subsequently, tissue sections containing lobule V near the midline in the medial-lateral plane were mounted onto glass slides, air-dried, and coverslipped with antifade Prolong mounting medium (Invitrogen). An LSM 710 Zeiss confocal microscope equipped with a 63X oil objective lens (NA 1.46) was used to collect confocal stacks using the ZEN imaging software. To unambiguously resolve dendrites of neighboring PCs in the parasagittal plane, the following image acquisition parameters were used: *x*, *y*-size of 0.22 μm/pixel; dimensions *x*: 224.7 μm, *y*: 224.7 μm; image size 1024 × 1024) and a *z*-step size of 0.25 μm.

Confocal image stacks were imported into Neurolucida software connected to a Wacom digital tablet and pen. Dendritic tree reconstructions were generated using interactive and manual tracing functions, in which points were inserted along each dendritic branch. The locations of these points and their coordinates in 3D space (x, y, and z) describe the geometric shape of each dendritic arbor. Somata were drawn using Neurolucida’s continuous tracing function, which inserted points along the contours of the somata, as visualized in each plane of the section (Supplementary Fig. 2).

### Computational modelling

The modelling workflow was developed using Python3 and NEURON 8.0^45^. To determine the maximum conductance value of each ionic channel, a genetic algorithm included in the Python package Blue Brain Python Optimization Library (BluePyOpt)^46^ was employed. Mouse and human morphological reconstructions and current clamp recordings were performed. Mouse PC models were first optimized given the wealth of prior electrophysiological and immunohistochemical data available for ionic channel localization and parameterization. The same conductance ranges (reported in Supplementary Table 3) obtained from mice were used as seeds to predict the properties of the human PC model. Finally, the human models were validated against corresponding electrophysiological recordings.

### Morphologies

PC morphological reconstructions (Fig. 2) were generated from mouse and human tissues using NeuroLucida. The mouse morphologies were curated to reduce irregularities in the dendritic trunks, while the human morphologies were used as provided (all morphologies are shown in Supplementary Fig. 3 and Supplementary Table 1). During model optimization, both mouse and human PC morphologies were equipped with axons obtained from a previous PC model^35^. Human PC AIS was maintained the same as in mice^53^ (no published data were available). The AIS length and diameter were consistent with measurements obtained from two human PC morphological reconstructions in which the AIS filling procedure was successful.

The dendrites of previous PC models^35^ were classified as either spiny or aspiny based on the dendritic properties of Guinea Pig PCs^29^. The parameters used for classification included dendritic length, dendritic diameter, total number of dendrites, and total length of dendritic trees. Here, we considered that the shape and morphology of PCs were conserved across species^54^ and adopted a similar strategy. The number of compartments belonging to each dendritic zone was determined considering the following parameters: 1) compartments with a diameter below 1.6 µm were defined as spiny dendrites, 2) compartments with a diameter between 1.6 µm and 3.3 µm were defined as part of the trunks, and 3) compartments with a diameter above 3.3 µm were part of the main trunks.

### Passive properties

The passive properties of mouse PC models were derived from those of the Guinea Pig PC model^27,28,35,36^. The axial resistance was maintained at R_a_ = 122 Ω·cm, whereas the membrane capacitance was set to C_m_ = 1 μF/cm^2^ for the soma and C_m_ = 2 μF/cm^2^ for dendritic compartments with a diameter larger than 1.6 μm. In agreement with previous models^27,28,35^, the compartments with a diameter below 1.6 μm received a variable C_m_ value to compensate for the spine surface (Eq.1).

1 $$C{m}_{{section}}=({11.510294} {\,\!}^{\wedge }(-1.376463* {section}.{diam})+2.120503)$$

This compensation was needed because spines were not included in the first version of the model (for models with spines, see the specific section below). According to previous models, the leak conductance was set to G_m_ = 0.003 S/cm^2^ for the soma and G_m_ = 0.0003 S/cm^2^ for the remaining compartments^27,28,32,35^. The leak-reversal potential for all compartments was E_leak_ = –61 mV.

The nodes of Ranvier and the myelinated compartments were taken from a previous PC cell model^35^ (see also (https://senselab.med.yale.edu/ModelDB/showmodel.cshtml?model=9848)^55^ with G_m_ = 5.60e-9 S/cm^2 and^ C_m_ = 1.87e-11 μF/cm^2^.

The same passive properties were also used for human PC models.

### Active membrane properties

The distribution of ionic channels, along with their reversal potential, calcium buffer, and pump densities, was obtained from previous PC models^35,36^. This choice proven effective; however, some adjustments were required to account for recent experimental data on mouse and human PCs.

The Kv1.1/2 potassium channels were previously distributed in the somato-dendritic compartments based on rat experimental data^56^, whereas the latest immunohistochemical data indicated the presence of the channel only on the main dendritic trunk^57,58^. The same was performed for another member of the Kv potassium family, Kv1.5, which was restricted to the soma and main trunks^57,58^ compared to previous reports that located the channel on the entire dendritic tree. The Kca2.2 potassium calcium-dependent channel was previously located in compartments above 1.6 µm, whereas in mice it can be found on the entire dendritic tree^59^. The calcium buffer was improved with the calcium protein calmodulin^60^ which was previously validated in two other cerebellar models^61,62^. The distribution of ion channels among the compartments, including the spines, is shown in a table in the supplementary material (Supplementary Fig. 4).

### Synaptic mechanisms

Based on previous PC models^32,36,61^, three synaptic zones were defined: (1) dendrites with a diameter between 0 and 0.75 µm were targeted by ascending axons (aa), (2), dendrites with a diameter between 0.75 µm and 1.6 µm were targeted by parallel fibers (pf), and (3) dendrites with a diameter above 1.6 µm were targeted by climbing fibers (cf). GABAergic synapses were distributed in compartments with diameters ranging from 0.3 to 1.6 µm^63^. The same ranges were used for the human models as there were no publications reporting specific information.

The membrane mechanisms for both AMPA and GABA receptors were the same as those described^36^, both with a peak synaptic conductance of 2600 pS. Recordings of human cerebellar AMPA, NMDA, or GABA receptor-mediated synaptic currents were not available. Synaptic receptors include the Tsodyks and Markram presynaptic vesicle cycle, 2D diffusion process, and Markov chain models to simulate postsynaptic receptors. Detailed information can be found in ref. ^64^.

### Feature extraction

Data features were extracted from the experimental traces in response to positive step current injection. The Blue Brain Project eFel library^65^ was used to extract the following features: AP amplitude, ISI_CV (coefficient of variation of the interspike interval (ISI_CV), afterhyperpolarization (AHP) depth (slow and absolute), AP width, voltage base, mean spike frequency, and spike count.

### Optimization of maximum ionic conductances

Maximum conductance parameters optimization was performed with BluePyOpt^46^, which leverages an Indicator-Based Selection in Multiobjective Search (IBEA) genetic algorithm (Supplementary Fig. 5, Supplementary Tables 2, 3, and 4). The algorithm generated a population of solutions (288 individuals) that were screened to obtain the best subset to prime the next generation. The fitness function was calculated for each objective as the difference between the features (average and standard deviation) of the experimental data and the corresponding parameters of the simulated traces.

Optimization was initially performed for a dozen generations on all morphologies to evaluate the impact of ionic channels on spontaneous firing and I/O relationships. This allowed us to determine the maximal and minimal conductance ranges that were valid for all the models. The final optimization was performed using these ranges, showing a near-zero fitness value within just 3–4 generations. Optimizations were run on the Piz Daint cluster (CSCS-Lugano) using eight nodes with 36 cores each, for a total of 288 cores. Simulations were performed using variable time steps, and model optimizations were performed with a fixed time step (0.025 ms). The smallest morphology (mouse morphology with 103 compartments) was optimized after 2 h, whereas the largest (human morphology with 1,880 compartments) was optimized after 19 h.

In accordance with previous cerebellar models^37,61,62^, instead of considering a limited number of best individuals, the entire final population was simulated using the BluePyOpt template extended with a custom-built MPI code (Mpi4py). This allowed us to distribute the simulations across the eight nodes (288 cores).

### Model validation workflow

The validation of both the mouse and human models was performed on Piz Daint (CSCS-Lugano) using a variable number of nodes depending on the number of validated individuals. The workflow is subdivided as follows:

The simulated voltage traces were analyzed to assess the presence of spontaneous firing activity (0 nA) and the I/O relationship (0.5 nA and 1 nA). Based on experimental data, a valid model should have spontaneous firing with a frequency between 5 Hz and 50 Hz, in compliance with known in vitro Zebrin+ PCs^66,67^. To complete the I/O relationship, the maximal frequencies for the 0.5 nA and 1 nA currents were set to 150 Hz and 200 Hz, respectively.

Sodium channels in the AIS are critical for AP generation^35,68^. The models were simulated for 1 s after the sodium channels in the AIS were removed. In this configuration, the models that had no ability to generate spontaneous APs were validated and passed on to the next phase.

The Input resistance (R_in_) was assessed using the NEURON voltage-clamp MOD file. Because it is not included in BluePyOpt, it was added to the template and configured to record the current for each simulation. The models were subjected to a 300 ms voltage clamp (−70 mV, -80 mV, −70 mV) with each step lasting for 100 ms. R_in_ was calculated automatically at the soma using the current recorded in the first step subtracted to the current recorded in the second step, and models were validated when R_in_ fell in the known experimental range. Since the absence of specific human data, R_in_ (14 MΩ) of the Guinea Pig model was taken as reference.

The last validation step required the addition of new compartment lists to the template to accommodate AMPA and used synaptic inputs instead of injected currents. Each model was provided with 50 excitatory synapses, one for each compartment belonging to the PF compartment list, which were chosen using a non-reinsertion method. We examined the results obtained from three bursts at 50 Hz, 100 Hz, and 200 Hz, each composed of 10 spikes. The bursts were delivered 5 times, with a delay of 1 s between, for a total simulation time of 5.5 s. Features were extracted from the last burst using a mobile time window to include only the spikes generated by the synaptic activity. The resulting voltage and current traces were stored as HDF5 files. The mouse and human models were validated using 9–10 spikes at 50 Hz, 4–6 spikes at 100 Hz, and 3–4 at 200 Hz (Supplementary Table 2).

Some of the validated models were randomly chosen and imported into a custom-made Python3/NEURON 8.0 script that replicated the responses and behaviors described in the previous sections. To reduce the computational requirement, the axon collateral was not included in the optimization; however, for consistency with previous models^35^, it was added afterwards, showing a negligible impact on the overall physiological properties.

Available human experimental data were used to validate human models. The dataset included a short portion of spontaneous firing, which was not sufficiently representative to calculate basal firing frequency and intrinsic spontaneous firing properties. Instead, positive current injections, consisting of three current steps, were quite effective and confirmed the requirement of three times more current to reach the mouse I/O relationship frequencies. At the same time, negative current injections required a stronger SAG than in the mouse data.

The simulations were performed using Python3, NEURON 8.0, at 32 °C, with a fixed time step (0.025 ms), on an AMD 1950x 16core/32threads.

### Spine morphology and modelling

To analyze the effect of localized synaptic patterns, spines were added to the models described above. Each spine consisted of two cylindrical compartments representing the head and neck. In mouse PC, the head was 0.35 µm long and 1 µm wide, and the neck was 0.7 µm long and 0.2 µm wide^69,70^. In human PCs, the head was 0.26 µm long and 1 µm wide, and the neck was 0.72 µm long and 0.18 µm wide (the measures were obtained from a morphological analysis of PCs reported in this paper). We set the parameters for the neck C_m_ = 3 μF/cm^2^ and the head C_m_ = 2 μF/cm^2^. Actually, the head C_m_ is reported to be 1.5–2 μF/cm^2^ in Guinea pigs^29^ but there are no corresponding estimates for mouse and humans. After checking that various combinations of C_m_ values had limited impact on spontaneous firing and synaptic responses, we set neck C_m_ above the usual value of 1 μF /cm^2^ to increase the filtering property of this compartment^71^ and to obtain appropriate burst/pause response properties (see Supplementary Fig. 7). Leak channels were placed in both the spine head and neck, whereas voltage-dependent ionic channels were placed only on the spine head, including Cav2.1 (P-type), Kca1.1 (BK), Kca2.2 (SK2)^59,72^, Kv4.3 (A-type), and Cav3.1 (T-type)^73,74^. The calcium channel Cav2.3 was experimentally proven not to be critical for intrinsic and synaptic responses; therefore, it was omitted^73^. Given the importance of calcium dynamics in controlling KCa1.1, the spines were endowed with a calcium buffer mechanism. The experimental spine density of 2 spines/µm^75–77^ was used both for mouse and human PC models (cf. the experimental data obtained from morphological reconstructions). When the models were simulated with active spines, C_m_ for the entire dendritic tree was set to 2 μF/cm^2^ regardless of compartment diameter^27,28,35,36^.

### Stimulus patterns with spiny dendrites

To activate a specific synaptic cluster, we developed a custom Python script defining ROIs by automatically dividing each morphology into square grids of 30 by 30 µm (900 µm^2^), containing spines belonging to aa, pf, and SC. The simulations were performed by applying the same synaptic patterns described above to 30% of the spines randomly chosen within a specific ROI^78^. In all simulations, the temperature was set to 32 °C and the integration time step was fixed at 0.025 ms. In a subset of simulations, background synaptic activity was generated by random activation of pf and SC synapses. In mouse PC models, 2,000 pf spines were stimulated at 1.1 Hz (random seed = 1) and 160 SC synapses were stimulated at 2.5 Hz (random seed = 0.4), yielding CV2 = 0.29^79^. In human PC models, 14,000 pf spines were stimulated at 1.1 Hz (random seed = 1) and 1000 SC synapses were stimulated at 2.5 Hz (random seed = 0.4) yielding CV2 = 0.26.

The computational load of the PC models with spiny dendrites required a single-blade cluster composed of two 32 cores AMD 7501 processors. The memory load during the longest simulation was about 10–15GB. The mouse simulations were better suited for running on 16 cores, whereas humans required 32 cores.

### Dendritic complexity index, synaptic independence, and impedance calculation

The Dendritic Complexity Index (DCI)^80,81^ yields a quantitative estimate of the extension and branching of a dendritic tree:

2 $${DCI}= 	 (\sum {branch}\,{tip}\,{orders}+N^\circ {branch}\,{tips}) \\ 	 * ({total}\,{dendritic}\,{length}/{total}\,{number}\,{of}\,{primary}\,{dendrites})$$

The values used in the equations are as follows: The branch tip order is an integer assigned to each terminal tip of the neuron’s dendrite and is equal to the number of branches emanating from the dendritic segment between a particular terminal tip and the cell body. The branch tips represent the total number of dendritic branches in the dendritic tree. The total dendritic length is the sum of the lengths of all segments. The total number of primary dendrites was the number of segments between the soma and the first branching point. In most mouse PC morphologies, the value is one, whereas in human PC morphologies it is two or three. These parameters were calculated using the NEURON simulator and TREE toolbox.

To evaluate synaptic independence, dendrites were divided into regions of interest (ROIs) containing 50 synapses and 25 randomly distributed SC synapses. Following a protocol used in a previous model, the stimulus pattern consisted of 50 pf synapses activated with five impulses at 100 Hz, followed by 25 SC synapses activated with three impulses at 142 Hz after 4 ms^36^. ROIs were stimulated in turn and voltage traces were recorded from all ROIs and from the main dendritic trunk, soma, AIS, and axons. Two ROIs were considered independent if activation of one generated a somatic response without altering the membrane voltage of the other (Supplementary figure 7).

Impedance was calculated with NEURON using the built-in impedance class. In both the human and mouse PC models, the soma was set as the target, and one dendrite every 20 was set as the source of the transfer function. Membrane voltage changes were elicited by injecting a sinusoidal current of 10 Hz into the dendrites. This allowed the calculation of the impendence at the injection site and the transfer function at other dendritic sites. Two compartments were considered independent when the transfer impedance was above the threshold level of 10 MΩ^82^ or when two dendrites were so distant that the stimulation had no reciprocal effect.

### Additional software for data analysis

Some of the results were analyzed using custom Python3 scripts and, for specific cases, MATLAB 2018b^83^ provided by the University of Pavia. The dendrograms were generated with NeuroM (BlueBrain Project (BBP) and the morphological properties were analyzed with a Matlab-based module, the TREES toolbox^84^. The fractal index was calculated using the Sierpinski triangle methods^85^ in the range of 0.01–1 (40 logarithmic scales). To provide the best possible resolution for the triangle method, the morphologies were plotted using Vaa3D, rescaled according to their somatic dimensions, and saved as images of 1024 × 1024 pixels. The 3D plots of the morphologies were generated using Vaa3D^86^. Measurements were taken from the automated analysis of dendrograms (NeuroM) and the TREES toolbox.

### Statistics and Reproducibility

The statistical properties of the data were represented using the mean and SD. A comparison between datasets was conducted using *t*-test statistics. Simulations can be reproduced using data and code uploaded to public repositories.

### Reporting summary

Further information on research design is available in the Nature Portfolio Reporting Summary linked to this article.

## Results

### Human and mouse PC morphological properties

Although rodent PCs have been intensely investigated^10–12,14,16,17,25,27–31,33,35,36^, the properties of human PCs remain largely unknown. Figure 1 shows the first recordings of human PCs in cerebellar slices obtained from fresh postsurgical specimens. Human PCs showed morphologies (Fig. 1a) and electroresponsive patterns (Fig. 1b) similar to those observed in rodents, although some striking differences were the greater dendritic tree and higher current needed to elicit action potentials in the somatic current-clamp recordings.

To investigate the implications of these differences, we performed a detailed comparison of human and mouse PCs using computational models. Overall, 19 mouse and 6 human PC 3D morphologies were reconstructed using fluorescence confocal microscopy (Fig. 2a) and used to generate dendrograms. (Fig. 2b; Supplementary Table 1), which recapitulates the dendritic structure in a graph made of segments stemming from branching points and can be used to reveal the architecture, hierarchies, and dependencies of dendritic branching. In both species, approximately 90% of the dendritic compartments were covered with spines (spiny dendrites). Moreover, in mice, 17/19 PCs have a single dendritic trunk, whereas the remaining 2/19 PCs have two dendritic trunks stemming from the soma^18,19^. In humans, 1/5 of PCs have a single dendritic trunk, 2/5 have two dendritic trunks, and 2/5 of PCs had three dendritic trunks stemming from the soma. Thus, human PCs had a much higher probability than mouse PCs (80% vs. 10.5%) to have multiple dendrites stemming from the soma (see also Supplementary figure 2).

The cumulative length of the dendritic tree was 2,782.59 ± 671.12 µm (n = 19) in mice and 20,166.96 ± 15,248.58 µm (n = 6) in human PCs, so that human PC dendrites were 7.24 times longer than mouse PC dendrites. In comparison, PCs of the guinea pig^29^, one of the biggest rodents, showed dendrites (9122.6 ± 1224.9 µm; n = 3) 3.27 times longer than mouse PCs^87,88^. The total number of segments, spiny dendrites, and aspiny dendrites were 2.9, 2.9, and 3.3 times larger in human PCs than in mouse PCs (Fig. 2c). The average spine density was similar in human and mouse PCs (2/µm) but since the total length of spiny dendrites was higher, the calculated total number of spines was 7.58 times higher in human than mouse PCs (Fig. 2c), a number that compares well with the increased number of spiny dendrites. As a whole, mouse PC dendrites had 5,064.47 ± 1,342.3 spines and human PC dendrites had 38,416.67 ± 29,209.88 spine (ranging up to 97,853 in the largest PC morphology). The dendritic branching orders had a broader range in humans (18–41) compared to that of mice (12–25) (Fig. 2d), reflecting the higher number of intermediate branches needed to demultiplex and scaffold a large number of terminal branches.

To quantify the impact of branching on PC architecture, we calculated the dendritic complexity index, or DCI^80,81^. This approach, based on purely morphological properties (Eq. 2), showed that human PCs had 27 times higher DCI compared to mouse PCs (average mouse = 4.7*10^6^ vs. average human = 1.3*10^8^) (Fig. 2c). Note that DCI in humans outperformed that in mouse PCs because of the higher number of branches and terminal branches as well as the total dendritic length, but not because of the higher number of primary dendrites (which appears in the denominator in Eq. 2).

We used fractal analysis to evaluate the scale invariance of the PC dendrites. The fractal dimension estimated with the Sierpinski triangle method^85^ was 1.72 ± 0.09 (n = 6) in human and 1.73 ± 0.03 (n = 19) in mice PCs. The fractal dimension was not different (p = 1, unpaired *t*-test) between the two species, suggesting that mouse and human PC geometries followed a similar scaling rule (Fig. 2c).

### Electrophysiological and biophysical properties of human and mouse PCs

**Fig. 3:**
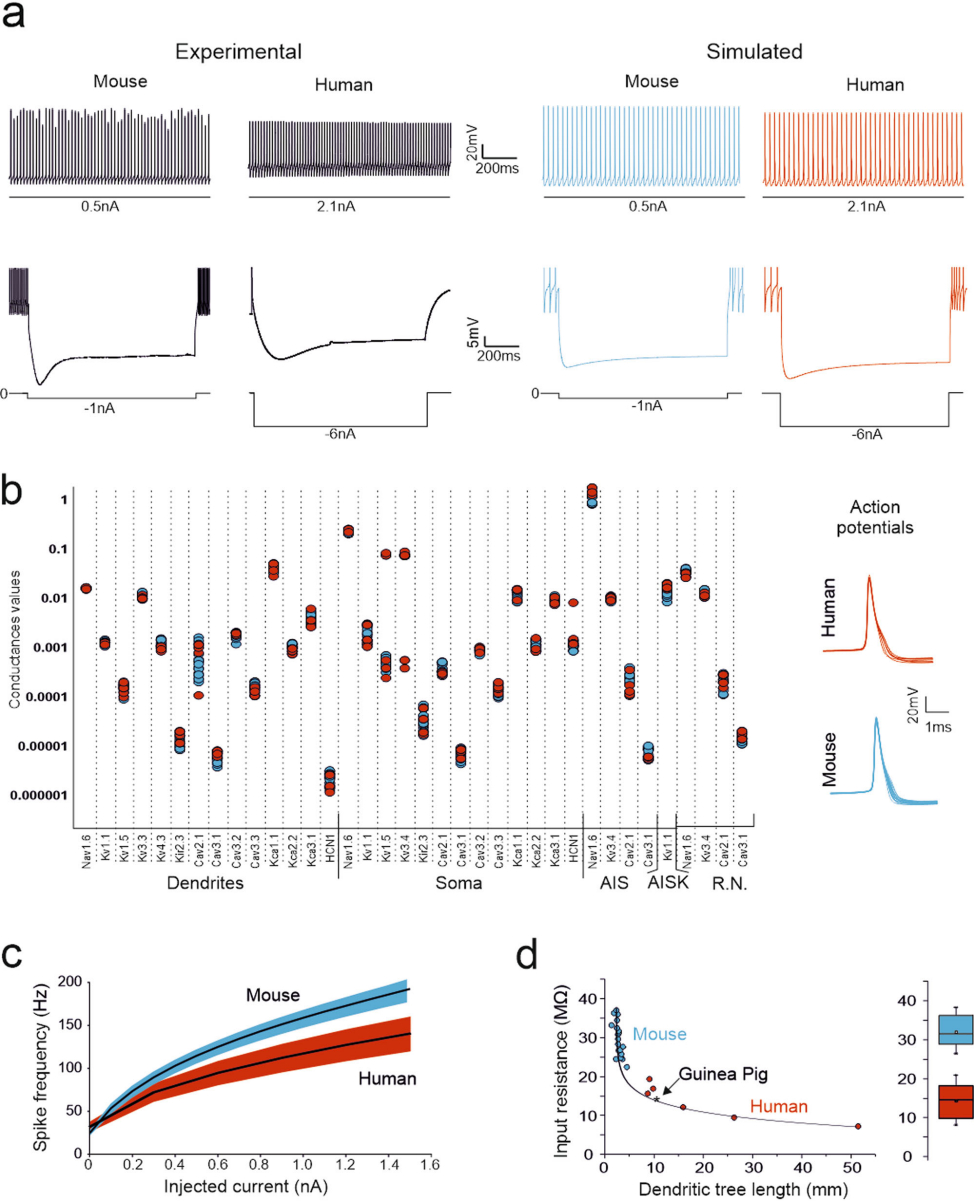
Electrophysiological recordings and model simulations. (**a**) experimental current-clamp recordings from a mouse and a human PC injected with positive (above) and negative (below) current steps. The two cells generated similar response patterns, but the human PC required higher current injection to attain the same responsiveness as that of the mouse PC.Simulations in a mouse and a human PC model injected with the same positive and negative current steps used in the experiments. Note that the mouse and human PC models generate similar responses, which closely correspond to those recorded experimentally. The higher current injection required by the human PC is due to the correspondingly lower R_in_. (**b**) The plot shows the maximum ionic conductance in the 19 mouse and 6 human PC models covered in this paper (blue for mouse and red for human PCs). Note that, for each ionic channel, data values are similar and tend to cluster irrespective of animal species. The traces on the right show action potentials in the mouse and human PC models. Except for minor differences in the AHP trajectory, action potentials were almost identical in the two species. (**c**) I-F relationships (mean ± s.d. represented as a colored area) for mouse and human PC models. The I-F slope was 2.05 ± 0.14 times higher in mouse than human models reflecting higher R_in_ (see panel d). (**d**) Dependence of R_in_ on dendritic length in the mouse and human PC models (R_in_ was estimated using voltage-clamp step protocols). R_in_ is inversely correlated with the length of the dendritic tree along an exponential decaying function, i.e., PCs with longer dendrites have smaller R_in_. Human PCs are in the right-hand branch of the curve, mouse PCs are in the left-hand branch, the guinea-pig PC^35,36^ is in the middle. The box plot shows statistics of R_in_ for all the mouse and human PC models.

To validate human PC models, we used PC current-clamp recordings from acute cerebellar slices (Figs. 1 and 3a). Only a limited number of human PC recordings have been obtained from fresh postsurgical cerebellar tissue preparations. Human PCs (n = 3) ex vivo showed rhythmic activity at rest before breaking into the whole-cell configuration. Once the whole-cell configuration was established, a constant hyperpolarizing current was injected to maintain the neurons at a subthreshold steady state. Subsequently, a series of depolarizing and hyperpolarizing current steps were injected to probe the neuron I-V and I-F properties relative to the steady state. It should be noted that this procedure, while stabilizing the recordings, did not allow us to assess the basal discharge frequency, which could be extrapolated by fitting the I-f curve (see below). In response to the depolarizing current steps, the PCs exhibited regular firing that increased in frequency with the injected current. In response to the hyperpolarizing current steps, the PCs showed sagging inward rectification (Fig. 3a). Similar voltage deflections and spike frequency changes were observed in the mouse PCs, although they required smaller current injections than the human PCs. The similarity in the spike response patterns and inward rectification suggests the engagement of a similar electroresponsive mechanism in mouse and human PCs.

### Computational models of human and mouse PCs

We used computational models to further analyze the PC intrinsic electroresponsiveness and fill the knowledge gap through principled rules based on neuronal biophysics^3^.

First, because humans appeared as rescaled versions of mouse and guinea pig PCs (see Figs. 1, 2), we assumed that the fundamental biophysical properties were also conserved. Thus, we generated both human and mouse PC models, starting from the PC model developed for guinea pigs, which currently represents the gold standard^27–29,35,36^.

Secondly, since the spike discharge was similar (see Figs. 1 and 3a), the same set of ionic channels was used in human and mouse as well as in the guinea pig PC models (Supplementary Table 3). This assumption was supported by the expression of equivalent channel genes in these species^89^. Ionic channels were placed in the neuronal compartments of human and mouse PC models, as it was done in the guinea pig PC model. The only exceptions were Kv1.1, which was restricted to the main dendritic trunk; Kv1.5, which was absent from the soma; and Kca2.2, which was distributed over the entire dendritic tree in humans and mice but not in guinea-pig PC models^58^.

Importantly, because the electrophysiological traces contain information about the contribution of all the engaged ionic channels, the maximum ionic conductances could be parameterized through an automatic optimization process^46^ based on the comparison between simulated traces and electrophysiological traces used as templates (see Methods). This methodology allowed us to optimally exploit the information contained in the human PC recordings. After parameter optimization, the human and mouse PC models did not show relevant differences in spike discharge and ionic conductance balance (a fact that was also true for the guinea pig PC model^35^); (Supplementary Table 4) (Fig. 3a, b), reflecting similar dendritic architectures and ionic channel complements. The spikes generated by these models during spontaneous firing exhibited nearly identical shapes in human and mouse PCs (Fig. 3b). the I-F relationship was also similar, but that of the mice was steeper (Fig. 3c). The average data for the spontaneous frequency and SAG amplitude are shown in Supplementary figure 6.

### Distinctive properties of human PC models

I-F relationships were generated for all human and mouse PC models using somatic step current injections (Fig. 3c; see Supplementary figure 6). The average I-F was similar, but the human PCs required 2.05 ± 0.14 times more positive current than mouse PC models to reach the same discharge frequencies (e.g., 100 Hz) (Fig. 3d). At 0-current injection, spontaneous firing frequency in mouse PCs (24.2 ± 3.4 Hz; n = 19) and human PC models (28.4 ± 8.0 Hz; n = 6) was statistically indistinguishable (unpaired t-test, p = 0.09) (Supplementary figure 6).

R_in_ was 30.73 ± 3.93 MΩ (n = 19) in mouse models and 13.01 ± 4.62 MΩ (n = 6) in human models (Fig. 3d). The R_in_ value decreased with the total length of the dendritic tree along a continuous exponential distribution, independent of species, with the guinea pig PC model lying in an intermediate position between the mouse (shortest) and human (longest) PCs (Fig. 3d). The R_in_ values of the mouse PC models corresponded closely with that reported experimentally^22^.

### Spontaneous firing and spike backpropagation in PC models

**Fig. 4:**
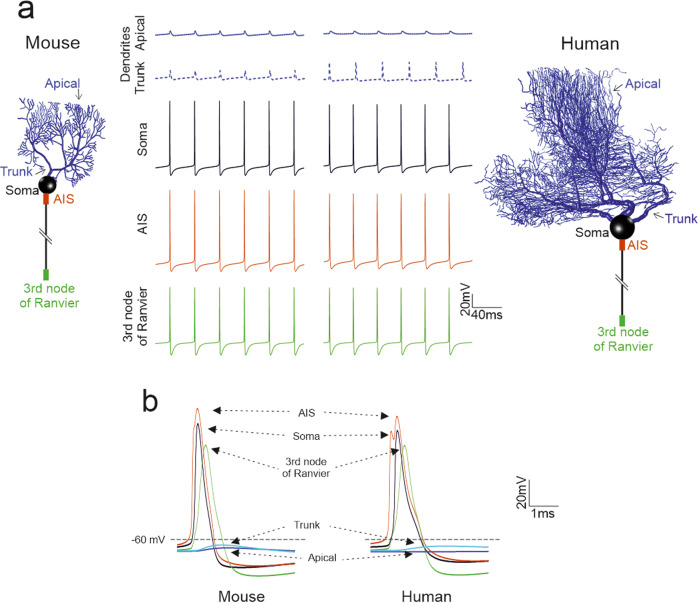
Spike propagation in PC models. (**a**) In all PC models, spikes are generated in the AIS and then propagate actively in the axon and passively in the dendrite, where they undergo a marked decrement and slowdown. Traces show the spontaneous spike discharge in different model compartments. The corresponding PC model morphologies are shown on the side (with the colors indicating the recording site). (**b**) Spikes taken from the same simulations as in A are overlay for AIS, soma, axon, aspiny, and spiny dendrites. Note that the AIS spike precedes those in any other compartment (same colors as in a).

The backpropagation of spontaneously generated APs through the dendritic tree showed remarkable similarities between the human and mouse PC models (Fig. 4a). APs were generated in the AIS where the AP reached its highest amplitude (40 mV). Then, the AP amplitude decreased when the spikes backpropagated to soma and dendrites, reaching –50 mV in the apical dendrites. These simulations reproduced typical PC property^11^ (Fig. 4b). The AP backpropagation also featured a delay that depended linearly on the distance from the soma. Therefore, in the model, the dendritic tree in human and mouse PCs behaved similarly to that in guinea pig PCs, that is, it acted as a filter, limiting AP backpropagation.

### Responses of PC models to randomly distributed synaptic inputs: burst-pause responses

To evaluate PC synaptic responsiveness, we assumed that the fundamental mechanisms of synaptic transmission were maintained across species in PCs, as well as in pyramidal neurons^7^. In addition, the burst/pause response elicited by synaptic inputs, which is observed in mice and monkeys^16^, should occur also in human PCs.

**Fig. 5:**
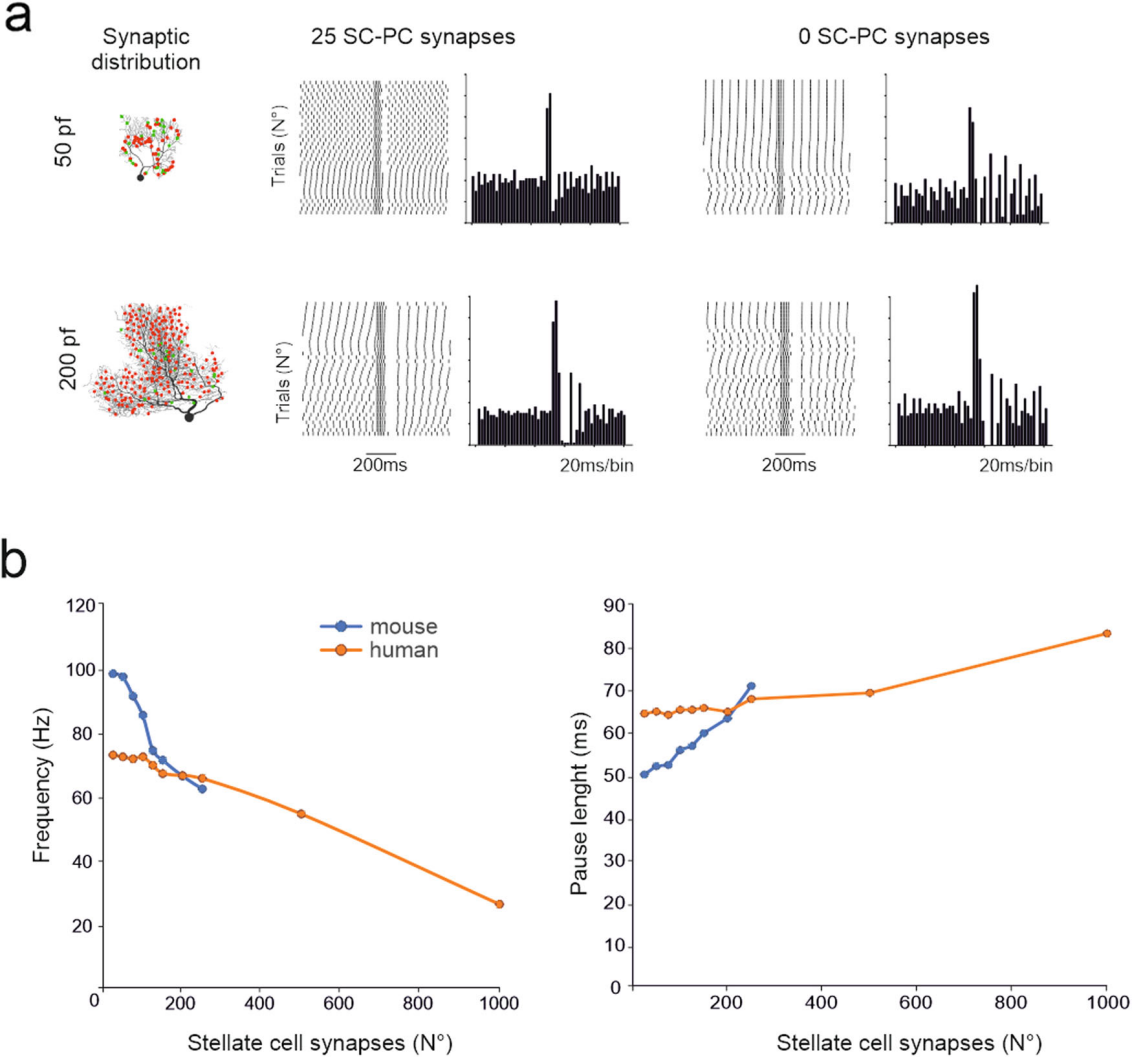
Burst/pause responses of PC models to synaptic inputs: the effect of synaptic inhibition. (**a**) In a mouse and a human PC model (same as in Fig. 4), 50 pf (red dots) and 25 SC synapses (green dots) were randomly distributed on the dendritic tree^36^. The stimulation pattern was composed of pf synapses activated with 5 impulses at 100 Hz followed by SC synapses activated with 3 impulses at 142 Hz after 4 ms^36^. Both models generate a burst/pause response that is evident in the raster plots and PSTHs. Note that the pause is more marked when inhibition is active. (**b**) The graphs show the modulation of burst and pauses by the number of active SC synapses for the same PCs shown in A. Both in mouse and human PCs, SCs curtail the burst and prolong the pause in a monotonic dose-dependent manner.

A burst/pause response was elicited in a human PC model using various combinations of excitatory and inhibitory synapses. An effective pattern capable of eliciting robust burst/pause responses in all human PC morphologies^33^ was composed of 50 pf synapses activated with five impulses at 100 Hz, followed by 25 SC synapses activated with three impulses at 142 Hz after 4 ms^36^ (Fig. 5a). With this combination of excitatory and inhibitory synapses, which were chosen randomly on the dendritic tree (see Methods), the burst showed a spike frequency increase from the basal level of ~100% (n = 19) in mice and by ~60% (n = 6) in human PC models. The burst frequency in human PC models reached that of mouse PC models by increasing the number of synapses from 50 to 200 (n = 6). Thus, the two models generated similar burst/pause responses if 4-times more pf were activated in human PCs to compensate for the lower R_in_. In general, the burst frequency decreased and the pause length increased with the number of active inhibitory synapses (Fig. 5b). A pause (i.e., a silent period longer than the basal ISI ~ 30 ms) was already present with 0 SC synapses, reflecting the involvement of an intrinsic after-hyperpolarizing mechanism following the burst^36^.

### Responses of PC models to localized synaptic inputs: dendritic independence

**Fig. 6:**
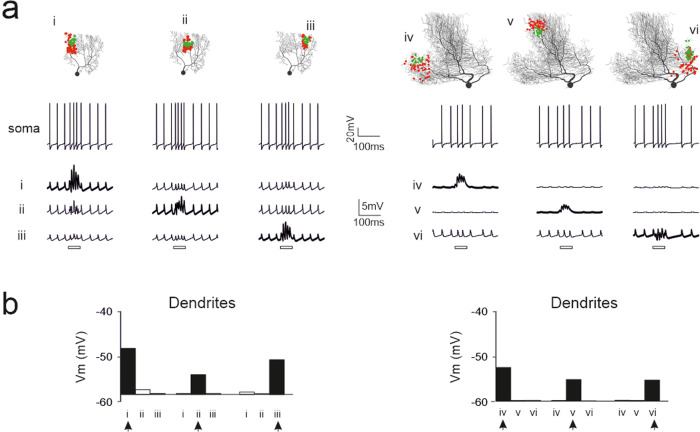
Responses of PC models to localized synaptic inputs: testing dendritic independence. (**a**) A mouse and a human PC model (same as in Fig. 5) are activated by 50 pf (red dots) and 25 SC synapses (green dots) placed on specific regions of interest (ROI) of the dendritic trees (i, ii, iii and iv, v, vi, respectively)^36^. The synapses were stimulated with the same number of impulses and frequency as in the previous image. For each stimulated ROI, the traces show burst/pause responses at the soma and local membrane potential changes in a compartment of the dendrites. The bars under the traces indicate the stimulation burst. The ROIs that are not directly stimulated show small variation in response amplitude, like spikes during spontaneous firing. (**b**) The histograms show membrane potential in a compartment inside each dendritic region averaged over burst duration. Note that membrane potential remarkably increases in the stimulated region (arrow) but remains almost unchanged with respect to basal level in the others.

To assess the impact of localized synaptic inputs, the dendritic tree of the PC models was stimulated on specific branches with the same synaptic activity pattern as defined above (50 pf with five impulses at 100 Hz and 25 SC with three impulses at 142 Hz delayed by 4 ms) (Fig. 6a). The activation of these branches effectively regulates spike/burst patterns in the soma. The independence of dendritic regions was assessed by stimulating one compartment and assessing its influence on the responses in other compartments (this was conveniently performed by defining ROIs; see Methods). Both human and mouse PC models showed large local responses in the stimulation ROIs, but little depolarization in other regions, demonstrating a remarkable degree of dendritic segregation. Notably, dendritic activation was able to generate a somatic response, which was then backpropagated and filtered (Fig. 6b; cf. ref. ^36^). These properties reflect PC coding and plasticity, as discussed in the discussion section.

### PC models with dendritic spines

**Fig. 7:**
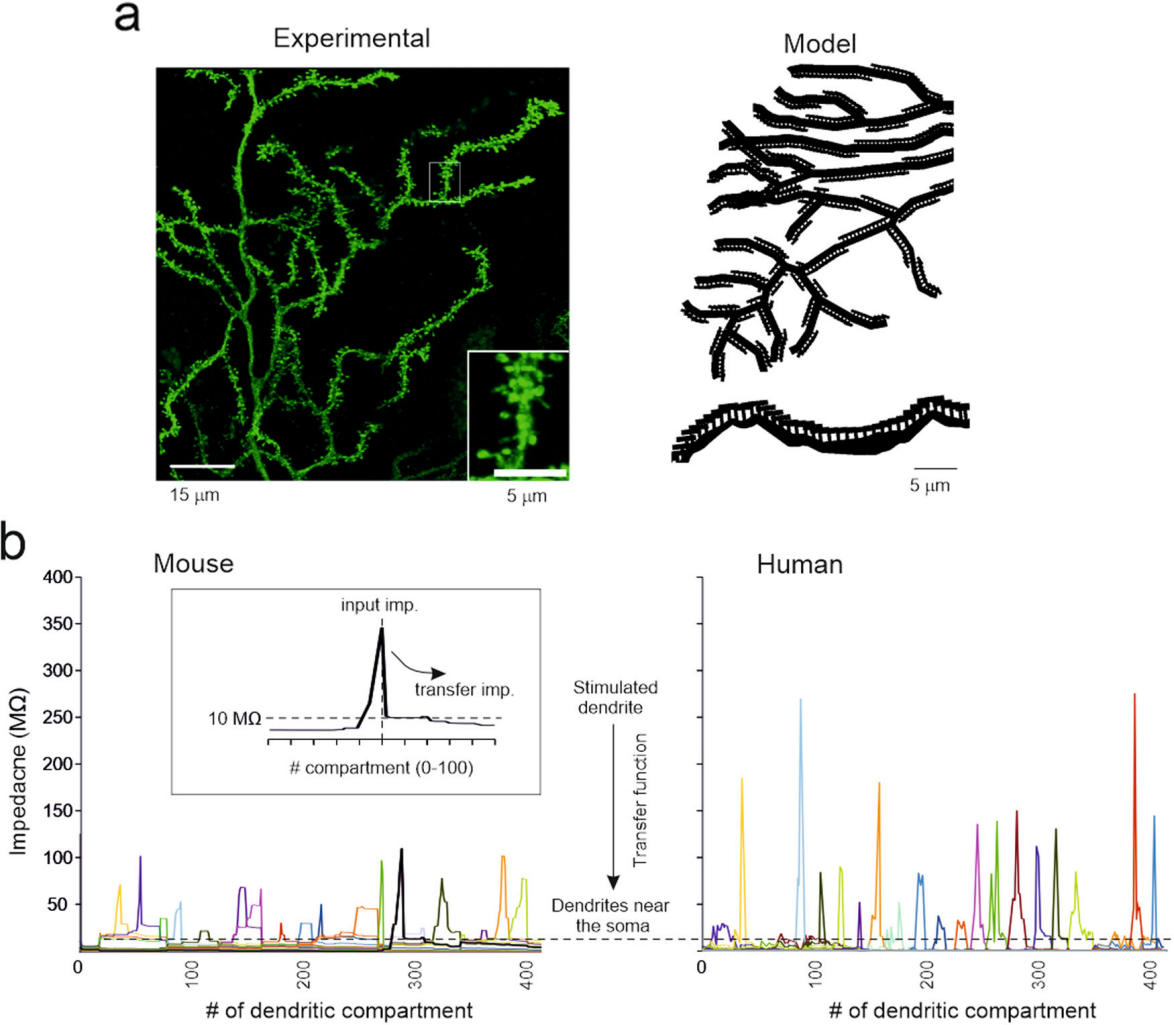
Testing dendritic independence with dendritic spines. (**a**) the panel shows a portion of the dendritic tree in a human PC intracellularly injected with Lucifer yellow. The inset shows spines at high magnification. The right panel shows the spines in the model drawn with NEURON graphics. The spine density is the same as in the experimental measurement, but spines are distributed on a plane instead of being displayed in 3D. (**b**) Examples of impedance traces calculated in a mouse and a human PC model. The X-axis reports the dendritic compartment indexed as in the dendrogram (the entire morphology is reported for the mouse PC while just ¼ of the morphology is reported for the human PC) and the colors identify responses to stimuli on different dendrites. A 10 Hz sinusoidal current was injected into a dendrite and the recorded voltage was used to calculate the transfer impedance (see inset and methods). Two compartments were considered independent when the transfer impedance was above the threshold level of 10 MΩ^82^ or when two dendrites were so distant that the stimulation had no reciprocal effect (similar to Fig. 6a). Note that the 10 MΩ threshold is crossed within 10–20 compartments from the stimulation site (see inset) and that impedance is higher in the human than mouse PC model, reflecting the greater length of the dendrites.

PCs express synaptic spines, which may affect the local electrical properties of dendrites^90^. Therefore, an advanced version of the PC models was developed using the spines. The spines were placed on the dendrites according to the density and distribution revealed by morphological analysis (Fig. 2c), and were endowed with active mechanisms according to the literature (Supplementary figure 4). Despite the much higher computational weight (spines increase neuronal compartments by approximately 100 times), models with spines were more realistic in terms of local postsynaptic mechanisms and were used to further investigate the ability of dendrites to process multiple synaptic input channels (Fig. 7a).

A 10 Hz sinusoidal current was injected into single dendrites, and the membrane potential change in the same and neighboring dendrites was used to calculate the transfer impedance (see Methods and Fig. 7b). Notably, the transfer impedance was higher in humans than in mouse PC models, reflecting the longer pathway that currents must travel beside the injection site. In both human and mouse PC models, the transfer impedance sharply decreased near the current injection site. When the transfer impedance was above the 10 MΩ threshold, the compartments were considered mostly independent. The operation was repeated at different dendritic locations, allowing to calculate an average value for the number of co-stimulated dendrites, which were 31.2 ± 22.2 (n = 37) in mouse and 14.7 ± 8.5 (n = 179) in human PC models. Given the total number of spiny dendrites ( ~ 250 in mice and ~750 in human PCs; Fig. 2c), the number of independent compartments were 8.0 in mouse and 51.1 in human PC models. This yields a 6.5 times higher number of computational elements in human PC models than in mouse PC models.

### PC models with dendritic spines

**Fig. 8:**
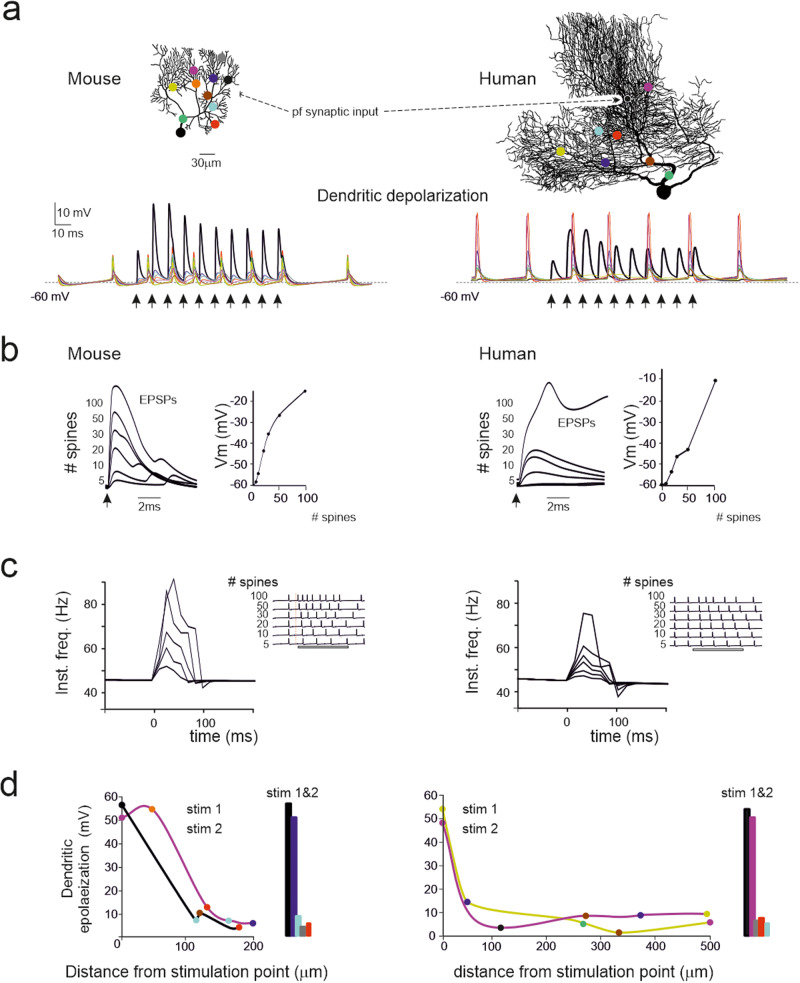
Synaptic excitation in spiny dendrites. **a** A mouse and a human PC model (the same as in Figs. 6–8) endowed with spines are activated by pf synapses (black dots) using bursts of 10 spikes at 100 Hz (arrows) on 50 and 89 spines, respectively. The traces show responses at the stimulation site (black) and at each other site (color coded). Note short-term facilitation in pf-PC EPSPs, a much larger response at the stimulated site than in any other sites, and synchronous back-propagated spikelets visible at all sites. (**b**) Dendritic EPSPs generated by activating an increasing number of pf synapses (second response in a train using the same impulse pattern used in A). The traces show EPSPs generated by an increasing number of spines and the plots on the right show the corresponding EPSP amplitude (the arrow indicates the stimulus). Note the sigmoidal shape of the activation curve (no response with 1 spine, rapid growth with an intermediate number of spines, tendency to plateau at more than 100 spines) both in mouse and human PC models. **c** Somatic spikes generated by activating an increasing number of pf synapses (same simulations as in **b**). The traces are spike trains (the arrows indicate the stimuli) elicited by an increasing number of activated spines. Note that the number of spikes elicited by the stimuli increases with the number of active synapses, as shown in the instantaneous frequency plots on the right. Note that in a–c the profile of EPSPs and spike responses is similar in mouse and human PC models, but the latter requires a larger number of active synapses. **d** PC model activation in the presence of synaptic noise. Synaptic stimuli were applied on two independent dendrites, either in isolation or combined, by activating 30% of the spines in each ROI with 5 spikes at 100 Hz on excitatory synapses and 3 spikes at 142 Hz on inhibitory synapses. The activity was recorded in the stimulated spines as well as in spines located elsewhere on the dendrites. The graphs show the net depolarization at a distance from the stimulation point. The histograms report the net depolarization with simultaneous stimulation in both sites (same color code as in panel **a**).

The synaptic responsiveness of the PC models was assessed by delivering an input burst (5 impulses at 100 Hz) to an increasing number of synapses confined to the same dendrite (Fig. 8a). Local EPSPs were slower in humans than in mouse PC models (Fig. 8b), probably because of the higher resistive and capacitive loads that increased the membrane time constant. The EPSP amplitude increased non-linearly, with comparatively smaller activation using just a few synapses, followed by rapid growth tending to plateau with more than 50 synapses. The crosstalk between the site of origin of the synaptic response and AIS was evaluated by measuring the changes in spike frequency at the soma (Fig. 8c). The activation of approximately five spines was sufficient to determine a sizeable change in the instantaneous spike frequency that progressively increased with the number of active spines. With > = 50 spines, the burst/pause complex appeared. In both species, the burst/pause response is generated by the full activation of a single terminal branch.

To test a real-life condition, the model was bombarded with a noisy input that slightly increased the basal frequency discharge by about 30% at the same time causing CV2 = 0.29 in mouse^79^ and CV2 = 0.26 in human PCs. Under these conditions, the dendrites demonstrated remarkable segregation of synaptic activity (Fig. 8d). The input decayed remarkably with distance, and in neighboring dendrites, depolarization was negligible. Interestingly, stimulating the two dendrites simultaneously did not have an impact on other regions. The two dendrites were chosen at opposite extremes of the dendritic tree in both the mouse and human PC models. In the human PC model, which had three independent dendrites, the stimuli on the leftmost and rightmost dendrites did not interfere with the intermediate dendrites, supporting the concept that multiple dendrites of human PCs can indeed operate as separate computational units.

## Discussion

This study addressed the functional and computational properties of the human cerebellar PCs. The PC structure and electroresponsiveness were similar to those of mouse PCs. However, human PCs have larger dendrites and higher dendritic complexity, which enables them to process more inputs than mouse PCs. Moreover, human PCs have multiple (2,3) dendrites stemming from the soma, which allow very effective segregation of synaptic inputs. Thus, human PCs can correlate with a larger variety of inputs than can rodent PCs, extending their computational capacity into a higher-dimensional space.

The morphological growth of PCs in humans does not affect either the length of terminal branches or spine density, but changes the number of dendritic trunks and the ramification needed to scaffold and demultiplex terminal branches. This makes the neuron geometry scale-invariant (or fractal), but simultaneously increases the dendritic complexity (see Eq. 2), and synaptic inputs. Interestingly, the presence of multiple dendritic trunks in human PCs has implications in climbing fiber connectivity and long-term synaptic plasticity^91^. For example, the high degree of segregation of PC dendrites implies local control of membrane potential and calcium concentration changes, suggesting that the three dendrites may be tuned almost independently by plasticity and then converge on the modulation of spike generation in AIS. The existence of PCs with multiple primary dendrites receiving multiple cf in adulthood was recently observed in both mice and humans, but with a higher probability in the latter^92,93^. Thus, dendritic rescaling implies an extension of the computational properties^29^, as suggested for CA1 pyramidal neurons^94^.

The intrinsic electroresponsiveness of human and mouse PCs is similar, with regular spike firing in the depolarizing direction, sagging inward rectification in the hyperpolarizing direction, and input-output functions (the I-F relationship) increasing sublinearly in the 100 Hz range^11,12,14,16^. Computational simulations showed that the I-F slope scales with R_in_ by a factor of approximately 2, reflecting the change in the PC size. In both human and mouse PCs, spikes are generated in the AIS and backpropagate through the dendritic tree, where they are filtered down to spikelets of a few millivolts, as observed experimentally in rodents^12,20,21^.

Simulations of dendritic activation suggested that the fundamental dendritic computational properties are similar in human and mouse PCs. A low release probability causes short-term facilitation^95^, so that sizeable dendritic responses occur when 2–3 parallel fiber spikes arrive in a short sequence. Moreover, the local response increases sigmoidally with the number of spines because of voltage-dependent calcium channel activation (cf.^36^). In this way, the co-activation of 3–5 neighboring spines is needed to generate EPSPs large enough to cause a detectable acceleration in the AIS spike discharge. The combination of local nonlinear amplification with short-term facilitation can effectively limit the transmission of sparse PF impulses, suggesting that the PC dendrites operate as high-pass filters, thereby increasing the signal-to-noise ratio. When a sufficient number of synapses are activated by repetitive parallel fiber inputs, a typical burst-pause response emerges, as documented from rodents to monkeys^16^. As shown in a guinea pig PC model, as well as in the human and mouse PC models, the burst-pause responses is generated by intrinsic membrane properties^35,36^ and is reinforced by inhibitory synaptic inputs. This opens an interesting scenario in which plastic modifications of the excitatory PF pathway and the inhibitory SC pathway can both contribute to regulating PC activation and control behavior in humans as well as in mouse PCs^96^.

The transfer impedance measured by injecting sinusoidal 10 Hz currents, which provides a functional estimate of the number of independent dendritic regions, yields the ratio of human/mouse = 6.5. Interestingly, this estimate converged toward the spine head ratio (human/mouse = 7.5), dendritic surface ratio (human/mouse = 5.5), and dendritic complexity ratio (human/mouse = 6.5). This suggests that the increased number of contacts is almost entirely transformed into effective combinations of input patterns that can regulate spike generation in the soma, akin to the linear encoding^26^ in a perceptron^25^.

The maximum computational capacity was calculated by considering the number of alternative states set up by the dendrites. The binary combinations are *K* = 2^*n*^, where *n* = 51 and *n* = 8 are the numbers of independent computational elements identified in the human and mouse PCs, respectively. This leads to C = 256 binary combinations for the mouse and C = 2.2*10^15^ binary combinations for the human PCs. Owing to the redundancy of dendritic combinations, some output spike patterns may be mutually indistinguishable on the temporal resolution scale of the neuron; therefore, C should be considered an upper limit. Ad hoc simulations may allow the calculation of combinatorial capacity in human and mouse PCs under more realistic assumptions; for example, that segments are not fully active or inactive or that independence is incomplete (ref. ^39–41^).

Rodent PC models have a long tradition^25,27,28,30–36^. In line with this, present mouse PC models have been heavily constrained by a rich electrophysiological dataset and a wealth of literature data used for construction and validation. Conversely, human PC models are uniquely based on 3D morphologies and somatic electrophysiological current-clamp recordings, which contain information about the ionic conductance determining intrinsic electroresponsiveness^46^. The expression of equivalent channel genes in PCs across species^89^ and the conservation of morphological features explain why the same set of ionic channels (with minor differences) can generate a similar intrinsic electroresponsiveness to somatic current injection. However, their properties may differ. For example, pyramidal neurons have been reported to express a shorter membrane time constant^4^ and enhanced EPSP amplification in humans than in mice to minimize the impact of their huge dendritic tree^7,51^. Moreover, the EPSPs generated by dendritic NMDA channels^51^ and HVA channels^6^ are larger in human pyramidal neurons, where they counteract dendritic filtering. Synaptic currents in human pyramidal neurons have been reported to have high conductance for optimal synaptic transmission and integration^51^. In another report, enhanced electrical compartmentalization of human pyramidal neuronal dendrites reduced their capacity to excite the soma^5^. It would be interesting to see whether some sort of adaptation also occurs in PC dendrites, in which the high resistive and capacitive loads slow down the EPSPs (e.g., see Fig. 8b) at odds with the requirements for fast processing on the millisecond scale of the cerebellar network^97^.

In conclusion, the main structural and electro-responsive properties of PCs have been conserved over almost 100 million years of evolution from rodents to humans^54^. However, human PCs exhibit increased dendritic complexity, resulting in a larger number of dendritic units that regulate neuronal output. This, in turn, causes a combinatorial explosion, increasing the computational capacity, whereas the segregation of inputs is enhanced by the larger number of dendrites stemming from the soma. The higher number of computations that can be carried out by humans compared to mouse PCs can be seen as a correlate of extended cerebellar connectivity. This, in humans, involves not only sensorimotor areas but also associative areas, which engage more than 80% of all the cerebello-cortical and cortico-cerebellar fiber tracts determined by MRI tractography^98^. Thus, the human PC operates as a multimodal perceptron combining a broad set of signals in the sensorimotor, cognitive, and emotional domains^99^ harmonizing the cerebellar response and integrating different aspects of behavior in a high-dimensional space. Whether specific adaptations of voltage-gated ionic channels, synaptic transmission, and passive membrane properties, which have been shown to improve the performance of human pyramidal neurons, also occur in human PCs remains to be assessed.

### Supplementary information

Supplementary Information

Description of Supplementary Materials

Supplementary Data 1

Reporting summary

## Data Availability

The data used for the box and whiskers plots, and certain graphs can be found in the Supplementary Data 1. The mouse morphologies can be found on neuromorpho.org

## Code availability

The computational models are available on eBRAINS and ModelDB (https://modeldb.science/267694)^100^.
