# Supplementary material for: Human Purkinje cells outperform mouse Purkinje cells in dendritic complexity and computational capacity
Source: Commun Biol. 2024 Jan 2;7:5. doi: 10.1038/s42003-023-05689-y (PMC10761885; doi:10.1038/s42003-023-05689-y)
Supplement: Supplementary file 1 — Supplementary Information [file 42003_2023_5689_MOESM1_ESM.pdf]

SUPPLEMENTARY MATERIAL

Supplementary Table 1: morphometric parameters of mouse and human PC used for modelling.

PC #	Dist dend	Prox_dend	Sodium dend	Tot dend	Tot dend len	Average len	Branchpoints	Sect/branch	Branch order	Branch angle
Mouse 1	197	20	1	218	2685.3	12.3	109	2	16	1.2
Mouse 2	319	16	2	337	3547.4	10.5	171	1.8	18	1.2
Mouse 3	214	16	1	231	2677.2	11.6	115	2.0	19	1.1
Mouse 4	218	17	3	238	2469.8	10.4	119	2	19	1.3
Mouse 5	320	17	2	339	3178.8	9.4	172	2.0	20	1.3
Mouse 6	258	11	1	270	2805.4	10.4	135	2	17	1.2
Mouse 7	288	19	6	313	3054.9	9.8	157	2.0	20	1.3
Mouse 8	172	11	2	185	2301.4	12.4	94	2.0	16	1.2
Mouse 9	178	11	2	191	2236.4	11.7	98	2.0	16	1.2
Mouse 10	433	26	1	460	4464.3	9.7	229	2.0	25	1.2
Mouse 11	82	8	2	92	1340.5	14.6	47	2.0	12	1.0
Mouse 12	310	24	4	338	3349.5	9.9	171	2.0	19	1.2
Mouse 13	124	14	1	139	1826.4	13.1	71	2.0	15	1.2
Mouse 14	228	14	1	243	2584.4	10.6	124	2.0	20	1.2
Mouse 15	261	15	3	279	2773.9	9.9	140	2.0	20	1.2
Mouse 16	257	22	1	280	2800.0	10.0	141	2.0	22	1.2
Mouse 17	341	17	2	360	3671.3	10.2	183	2.0	17	1.2
Mouse 18	223	14	5	242	2470.8	10.2	124	2.0	20	1.2
Mouse 19	218	15	2	235	2631.4	11.2	118	2.0	18	1.1
Human 1	1678	93	35	1806	51426.7	28.5	925	2.0	42	1.3
Human 2	354	33	6	393	9817.3	25.0	199	2.0	28	1.2
Human 3	303	30	2	335	9019.7	27.0	172	2.0	18	1.2
Human 4	285	35	7	327	8680.9	27.0	164	2.0	18	1.2
Human 5	526	26	8	560	15873.3	28.3	294	2.0	22	1.3
Human 6	1145	40	52	1237	26183.8	21.2	642	2.0	39	1.2

Table 1. Morphometric parameters obtained from NEURON (columns 1-6) and Tree toolbox (columns 7-10) in each one of the reconstructed neuronal morphologies used for modelling.

Dist dend: Number of spiny terminal dendrites
Prox dend: Number of aspiny proximal dendrites
Sodium dend: Number of aspiny proximal dendrites endowed with sodium channels
Tot dend: Total number of dendritic sections
Tot dend len: Total dendritic length in μm
Average len: Average length of dendritic sections
Branch points: Total number of branch points
Sect/branch: Number of sections for each branch point
Branch order: Order of the branches obtained using the tree toolbox
Branch angle: Average angle (in degrees) of the section departing from a branch point

Supplementary Table 2: optimisation results of mouse and human PC models.

Morphology	Individuals	Valid I/O	% I/O	Valid AIS	% AIS	Valid Synaptic activity	% Syn
Mouse 1	576	491	85.24	491	85.24	383	66.49
Mouse 2	576	531	92.18	531	92.18	89	15.45
Mouse 3	576	384	66.66	384	66.66	168	29.16
Mouse 4	576	485	84.20	485	84.20	128	22.22
Mouse 5	576	567	98.43	565	98.09	394	68.40
Mouse 6	576	522	90.62	522	90.62	457	79.34
Mouse 7	576	428	74.30	428	74.30	243	42.18
Mouse 8	576	340	59.02	340	59.02	107	18.57
Mouse 9	576	569	98.78	567	98.43	17	2.951
Mouse 10	576	221	38.36	221	38.36	68	11.80
Mouse 11	576	538	93.40	233	40.45	210	36.45
Mouse 12	576	542	94.09	542	94.09	7	1.215
Mouse 13	576	354	61.45	354	61.45	60	10.41
Mouse 14	576	394	68.40	394	68.40	124	21.52
Mouse 15	576	393	68.22	393	68.22	68	11.80
Mouse 16	576	565	98.09	565	98.09	350	60.76
Mouse 17	576	114	19.79	114	19.79	41	7.118
Mouse 18	576	455	78.99	455	78.99	247	42.88
Mouse 19	576	519	90.10	519	90.10	380	65.97
Total	10944	8412	76.86	8103	74.04	3541	32.35
Human 1	576	509	88.36	509	88.36	246	42.70
Human 2	576	404	70.13	145	25.17	70	12.15
Human 3	576	499	86.63	482	83.68	458	79.51
Human 4	576	575	99.82	217	37.67	22	3.81
Human 5	576	559	97.04	552	95.83	128	22.22
Human 6	576	512	88.88	458	79.51	15	2.60
Total	3456	3058	88.48	2363	68.37	939	27.17

Table 2. The table illustrates the optimization and validation process.

Individuals: total number of individuals in the last generation of optimization
Valid I/O: number of individuals with a correct Input Output relationship
% I/O: percentage of individuals valid for the Input Output relationship
Valid AIS: number of individuals valid for the absence of intrinsic activity if the sodium channels are missing from the Axon Initial Segment.
% AIS: percentage of individuals valid for the absence of intrinsic activity if the sodium channels are missing from the Axon Initial Segment.
Valid Synaptic activity: Number of individuals validated for the synaptic activity.
% Syn: Percentage of individuals validated for synaptic activity.

Supplementary Table 3: ionic channels and maximum conductances ranges.

Ionic channel type	Location	Conductance ranges (mA/cm^2)
Kv3.3	0 µm<= Dendrites <= 1.6 µm	0.009 - 0.013
Kv4.3		0.0008 - 0.001
Cav2.1		7.5e-3 - 2e-2
Cav3.1		4e-6 - 8e-6
Cav3.2		0.00085 - 0.002
Cav3.3		0.0001 - 0.00017
Kca1.1		2.5e-2 - 4e-2
Kca2.2		7e-4 - 1e-3
HCN1		0.000001 - 0.0000032
Kv1.1	Dendrites >= 1.6 µm	0.001 - 0.0013
Kv1.5		0.9e-4 - 1.5e-4
Kir2.3		0.000008 - 0.00002
Kca3.1		0.0025 - 0.006
Nav1.6	Dendrites >= 3.3 µm	0.0145, 0.016
Nav1.6	Soma	0.21 - 0.25
Kv1.1		0.001 - 0.003
Kv1.5		3.5e-4 - 5e-4
Kv3.4		0.07 - 0.1
Kir2.3		0.000015 - 0.00007
Cav2.1		2.5e-4 - 5e-4
Cav3.1		4e-6 - 9e-6
Cav3.2		0.0006 - 0.001
Cav3.3		0.00009 - 0.00015
Kca1.1		0.008 - 0.015
Kca2.2		8e-4 - 1.5e-3
Kca3.1		0.007 - 0.011
HCN1		0.001 - 0.009
Nav1.6	AIS	1.75 - 2
Kv3.4		0.008 - 0.011
Cav2.1		1e-4 - 4e-4
Cav3.1		5e-6 - 1e-5
Kv1.1	Para AIS	0.008 - 0.02
Nav1.6	Nodes of Ranvier	0.025 - 0.04
Kv3.4		0.01 - 0.015
Cav2.1		1e-4 - 3e-4
Cav3.1		1e-5 - 2e-5

- Ionic channel type: ionic channel type based on an international classification [1]
- Location: the location along the morphology were the ionic channels were placed.
- Conductance ranges (mA/cm^2): The parameter ranges were used as priors for the optimization process and were the same both in mouse and human PC models.

Supplementary Table 4: ionic channels maximum conductance following optimization.

Ionic channel type	Location	Max conductance (mA/cm^2)
Kv3.3	0 µm<= Dendrites <= 1.6 µm	0.01065 ± 0.0012937
Kv4.3		0.00102 ± 0.0001928
Cav2.1		0.00099 ± 0.0004428
Cav3.1		7.11566E-06 ± 7.32095E-07
Cav3.2		0.00181 ± 9.11506E-05
Cav3.3		0.00014 ± 3.17121E-05
Kca1.1		0.03915 ± 0.0085483
Kca2.2		0.00093 ± 0.0001712
HCN1		2.05563E-06 ± 6.62379E-07
Kv1.1	Dendrites >= 1.6µm	0.00122 ± 3.1379E-05
Kv1.5		0.00012 ± 2.1532E-05
Kir2.3		1.23279E-05 ± 4.28247E-06
Kca3.1		0.00370 ± 0.0009820
Nav1.6	Dendrites >= 3.3	0.0151 ±0.0005024
Nav1.6	Soma	0.21720 ± 0.0236269
Kv1.1		0.00174 ± 0.0007324
Kv1.5		0.00048 ± 0.0001599
Kv3.4		0.07377 ± 0.0054591
Kir2.3		3.56706E-05 ± 2.15094E-05
Cav2.1		0.00035 ± 8.84837E-05
Cav3.1		6.95137E-06 ± 1.79487E-06
Cav3.2		0.00090 ± 9.3038E-05
Cav3.3		0.00012 ± 1.93729E-05
Kca1.1		0.01186 ± 0.0025027
Kca2.2		0.00120 ± 0.0003136
Kca3.1		0.00861 ± 0.0015543
HCN1		0.00225 ± 0.0027538
Nav1.6	AIS	1.21748 ± 0.1464244
Kv3.4		0.00981 ± 0.0008169
Cav2.1		0.00022 ± 9.69215E-05
Cav3.1		6.50376E-06 ± 1.59735E-06
Kv1.1	Para AIS	0.01334 ± 0.0039573
Nav1.6	Nodes of Ranvier	0.02938 ± 0.0028718
Kv3.4		0.01185 ± 0.0012497
Cav2.1		0.00018 ± 6.80297E-05
Cav3.1		1.53709E-05 ± 3.31895E-06

- Ionic channel type: ionic channel type based on an international classification [1]
- Location: the location along the morphology where the ionic channels were placed.
- Max conductance (mA/cm^2): Conductances ranges obtained after optimization in mouse (n=3) and human (n=3) PC models.

Supplementary Figure 1: intracellular injection and reconstruction of human PCs

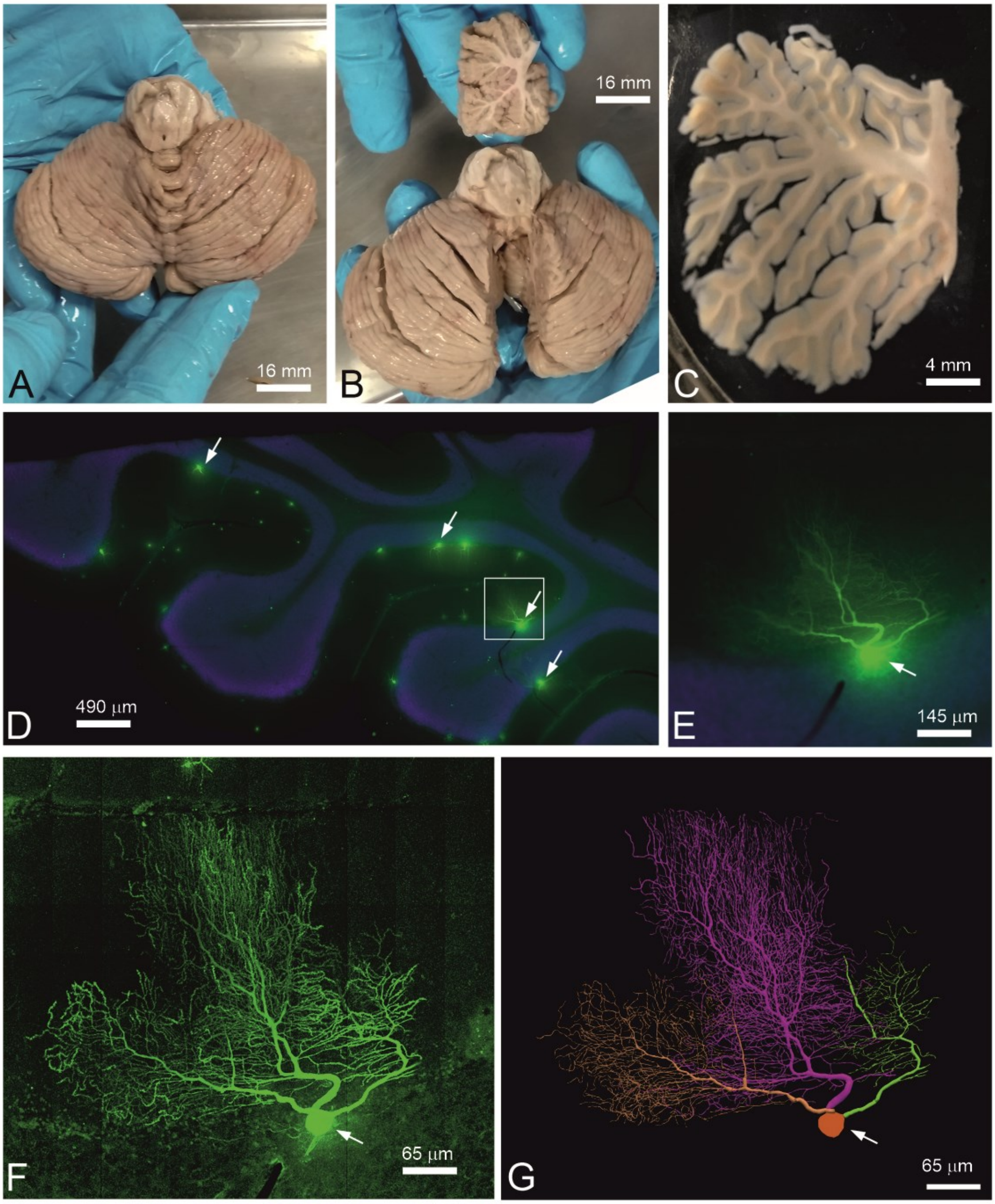

A and B show a dorsal view of the human cerebellum (AB6 case) and a tissue block (B top) obtained from the vermis region of the anterior and posterior lobes. C shows an example of the vibratome sections that were used to intracellularly inject PCs with Lucifer yellow. D, low power conventional fluorescence photomicrograph through a cerebellar folia showing examples of PCs (arrows) intracellularly injected with Lucifer yellow. E show an intracellularly injected PC (arrow), corresponding to the squared zone in D, that was subsequently scanned by confocal microscopy (shown in F as a z-projection image) and reconstructed with Neurolucida software (G). Scale bar, shown in G, indicates 16 mm in A and B, 4 mm in C, 490 µm in D, 145 µm in E, and 65 µm in F and G.

Supplementary Figure 2: counting primary dendrites on human Purkinje cells.

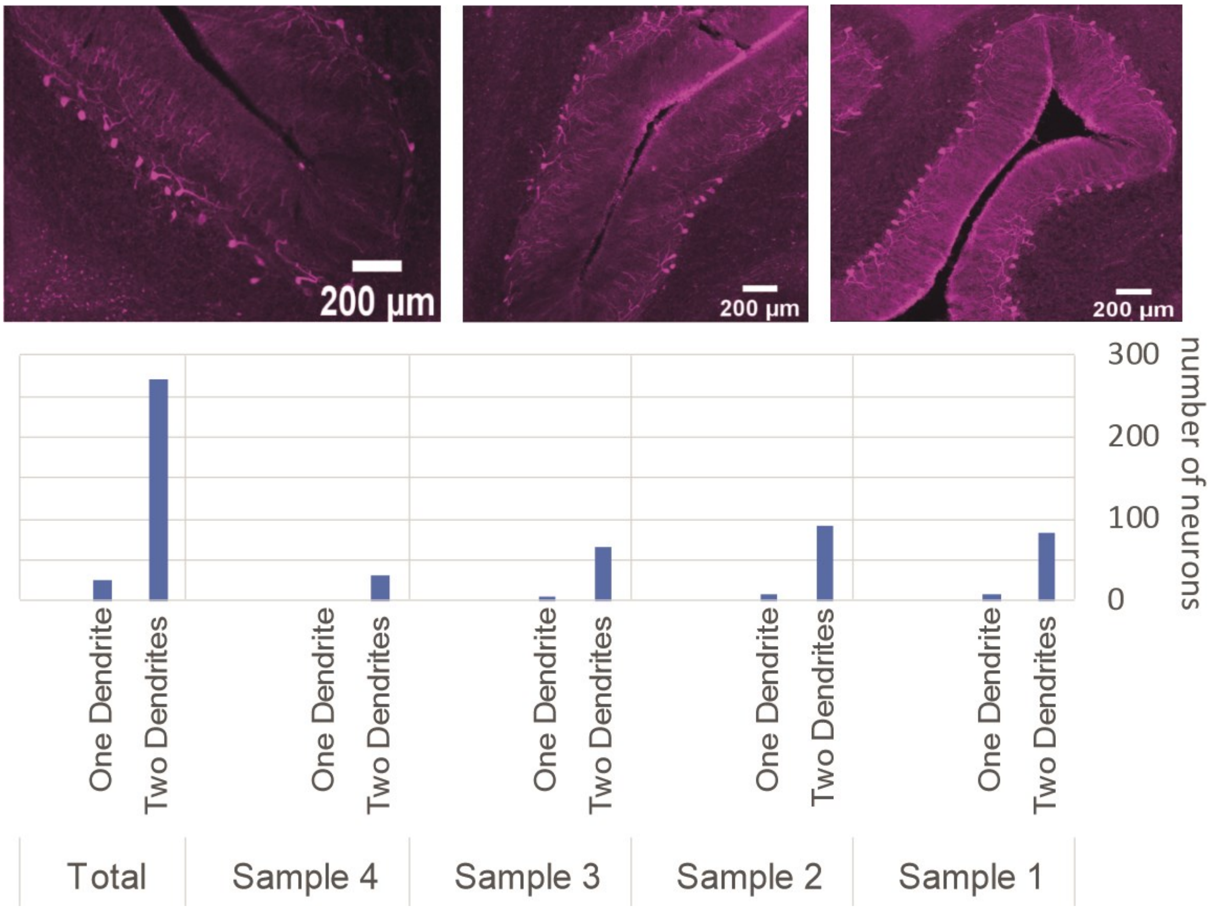

Top: representative examples of human cerebellar cortex tissue sections with stained PCs. Primary dendrites can generally be clearly seen when the sectioning angle matches the translobular plane. Bottom: histogram summarizing results from 4 different tissue samples; three additional tissue samples were excluded because PC morphologies were too far degraded before tissue fixation and staining did not show primary dendrites clearly. The figure shows sections of 30 µm, 40 µm and 50 µm respectively with 1:5000 primary antibody concentration. Human post mortem male brain of age 61 fixed with 4% PFA.

METHODS: Fixed human brain tissue specimens were obtained through the NIH NeuroBioBank following a request for a preliminary study on this topic. Specimens were prepared at various times post mortem and using either paraformaldehyde or formalin as fixative. Following visual inspection to determine the best possible translobular sectioning plane, a small piece was cut from each specimen and washed in PBS. A randomly chosen subset of these tissues was placed in 30% sucrose in PBS, embedded in O.C.T. compound (brand) and cooled to -80°C before being sectioned to 20 - 50 um thickness using a cryostat, while the remaining tissues were sectioned to 50 - 100 um thickness using a vibratome. Tissues were collected and stored free-floating in PBS until staining, which was performed using a primary antibody targeting the calbindin-D28K protein (locally specific to Purkinje neurons; Swant) and a matching far-red secondary antibody (donkey-anti-rabbit-AlexaFluor-647). The general procedure was as follows: first, the tissues were rinsed 4 times in PBS for 4 minutes, and then incubated in a blocking buffer containing Normal Donkey Serum, PBS and 0.3% Triton x-100.

400ul of blocking buffer for 60 minutes at room temperature. Slices were then incubated with the primary antibody (diluted 1:2000 or 1:5000 in the blocking buffer) overnight at 4C°. On the next day the sections were rinsed 4 times 4 minutes with PBS, and then incubated in blocking solution with the secondary antibody (donkey-anti-rabbit-AlexaFluor-647) added at a concentration of 1:1000 for 90 minutes at 4C° in the dark and finally rinsed in PBS 4 times for 4 minutes and stored in PBS Azide (no more than 36 hours) until mounting.

Slices were mounted on glass microscopy slides using fine brushes, taking care that folia would be lying down flat and not get twisted. Once dried thoroughly, a few drops of mounting medium (ProLong™ Diamond Antifade Mountant or ThermoScientific PermaFluor Aqueous Mounting Medium) were placed on each slide and the slides were covered using coverslips and allowed to cure according to the mounting medium's manufacturers' recommendations (30min - 48hrs). After the curing period, the slices were imaged using an Olympus Bright Field Microscope. Areas of interest were identified under 4x magnification, and subsequently captured using a 40x magnification objective. Images were viewed and post-processed to adjust brightness and contrast using ImageJ [2], and 8 images with particularly clear single PN layers in the right orientation were selected for further analysis; only sections in which primary dendrites could be counted for >80% of PNs were used. Altogether, 350 PNs were examined across 4 different tissue samples, and 569 primary dendrites were counted on 297 PNs. The resulting counts were logged per image-section and from these counts, the percentage of PNs with multiple primary dendrites was calculated.

Supplementary Figure 3: mouse and human morphologies.

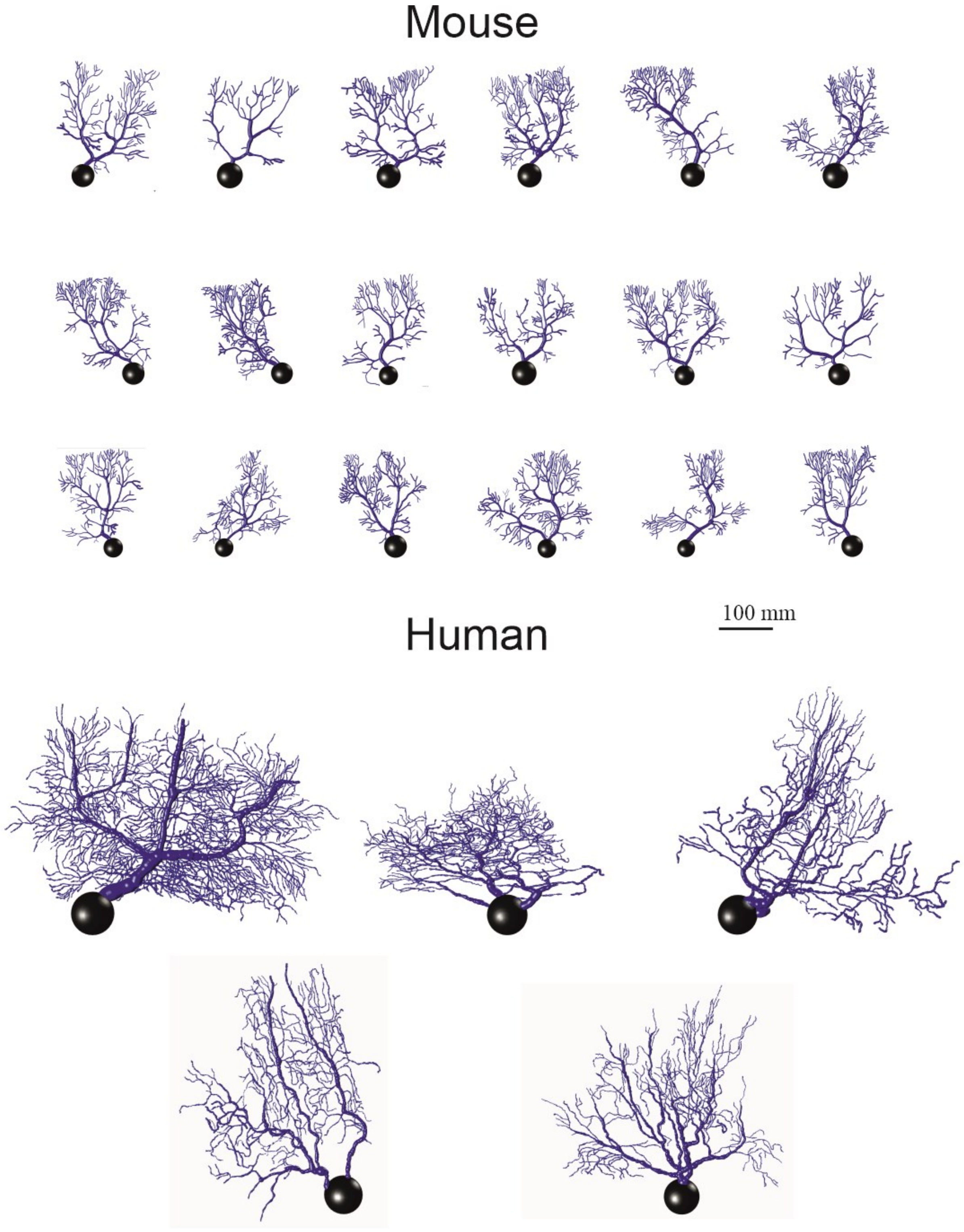

The figure illustrates the entire dataset of morphologies used in the construction and validation of the PCs models.

Supplementary Figure 4: ionic channel types and distribution

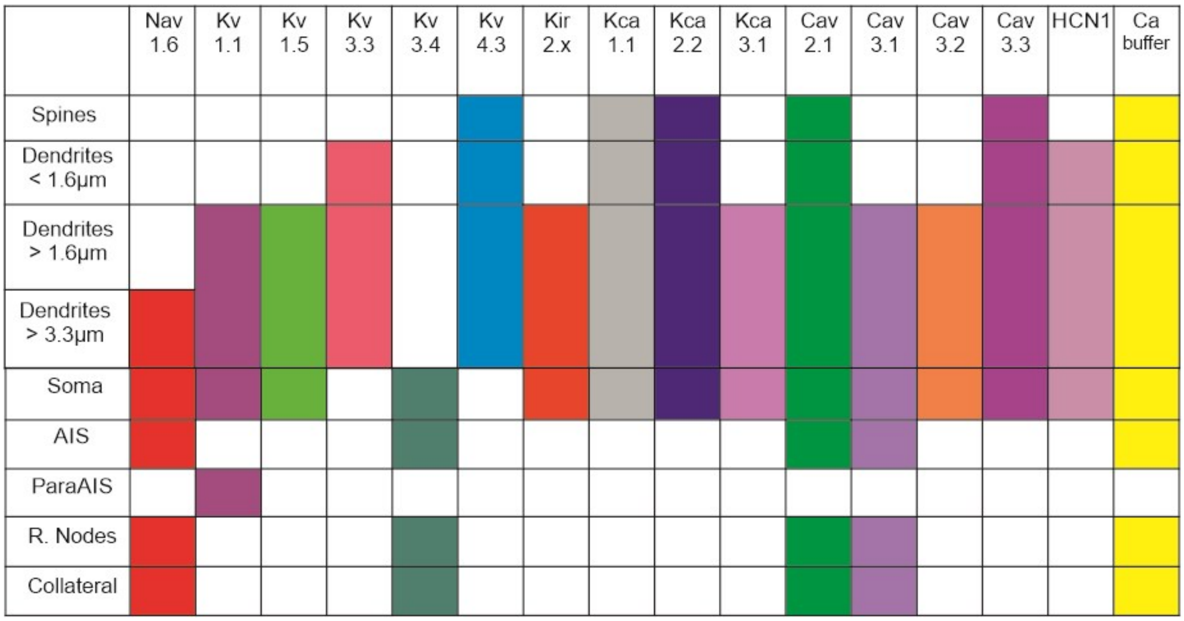

The figure illustrates the distribution of ionic channels and Ca buffer in the PC models. The ionic channels mechanism were taken from a previous model [3,4] and updated to reflect new experimental data. The KCa2.2 channel was distributed over the entire dendritic tree, whereas Kv1.1 was restricted to proximal dendrites and Kv1.5 to proximal dendrites and soma. Specific ionic channels were inserted in spines according to literature.

Supplementary Figure 5: optimization process

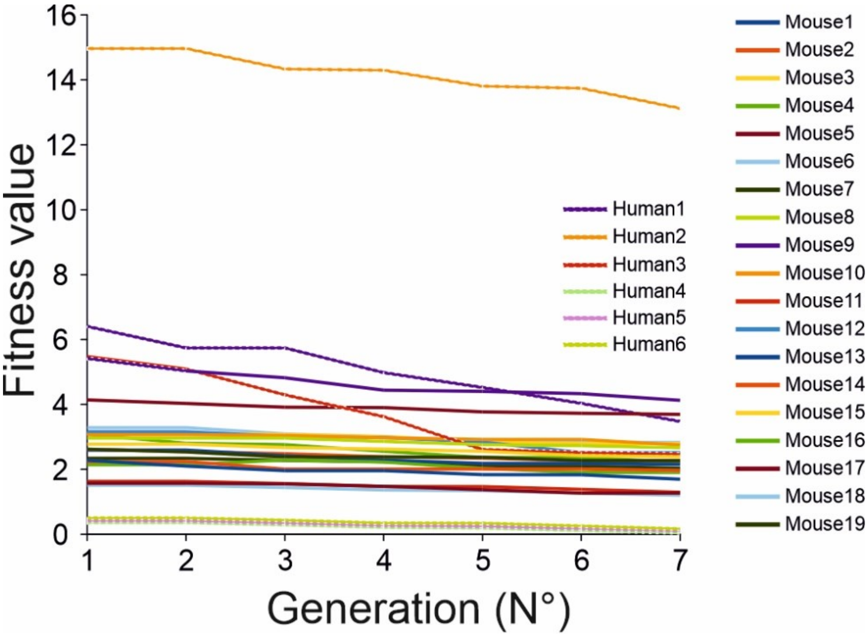

The panel shows the progress of optimization through subsequent generations. The fitness values for the 25 PC models tend toward zero in just 7 generations, showing improved matching between experimental data and modelling results.

Supplementary Figure 6: ramp current injection

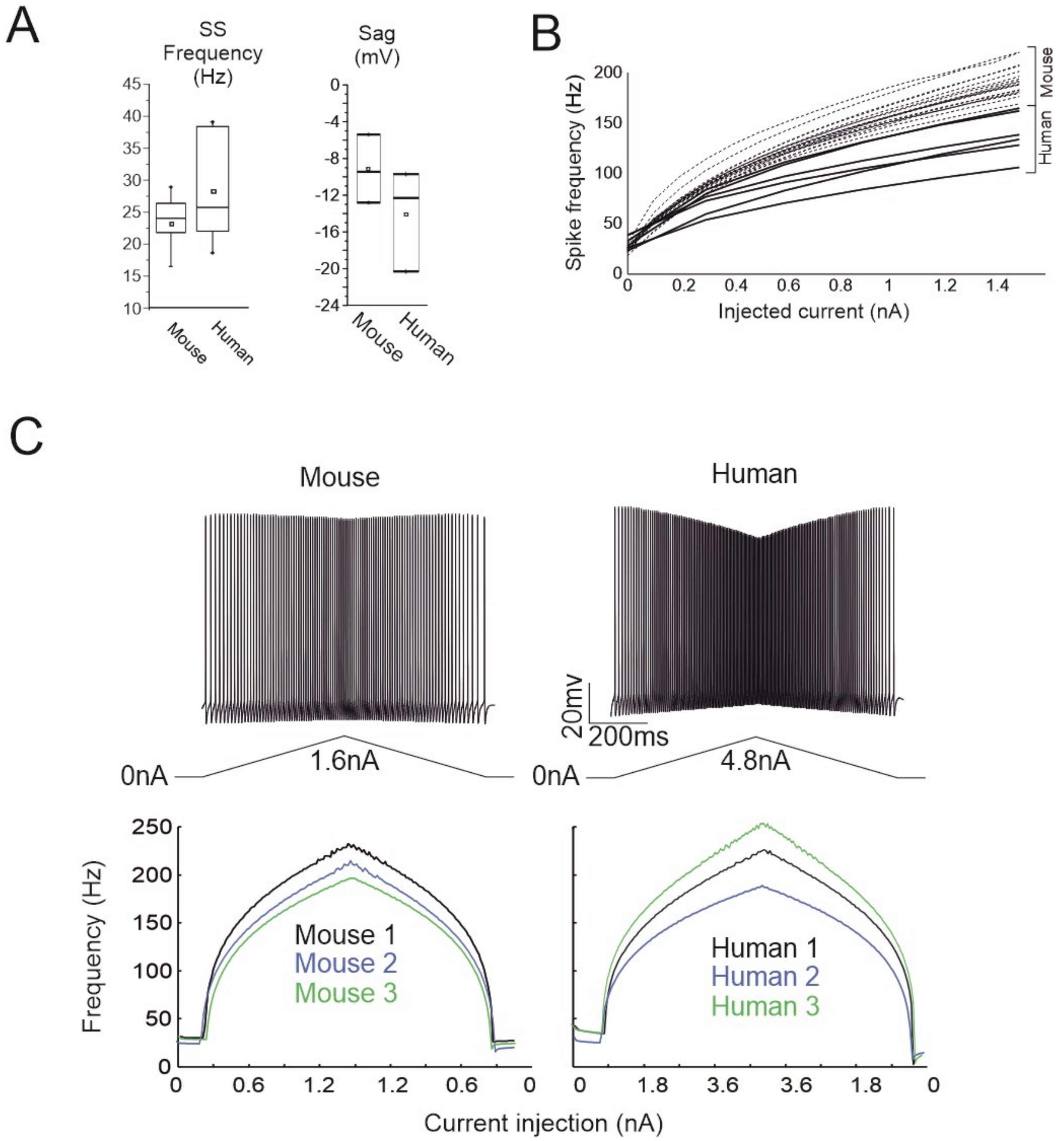

(A) The box plot shows statistics of spontaneous firing frequency for all the mouse and human PC models. The square at the center of each box define the mean, the line in the box define the median and the x define the Outliers

(B) I-F relationships for all PC models covered in this study. The I-F relationships shows similar shape in human and mice PCs.

(C) A ramp current injection, from 0 to 1.6 nA in mouse and from 0 to 4.8 nA in human PC models, reproduced a typical PC voltage response [5]. A marked frequency reduction appears in the falling branch of the ramp, causing an asymmetric instantaneous frequency profile.

Supplementary Figure 7: spines Cm

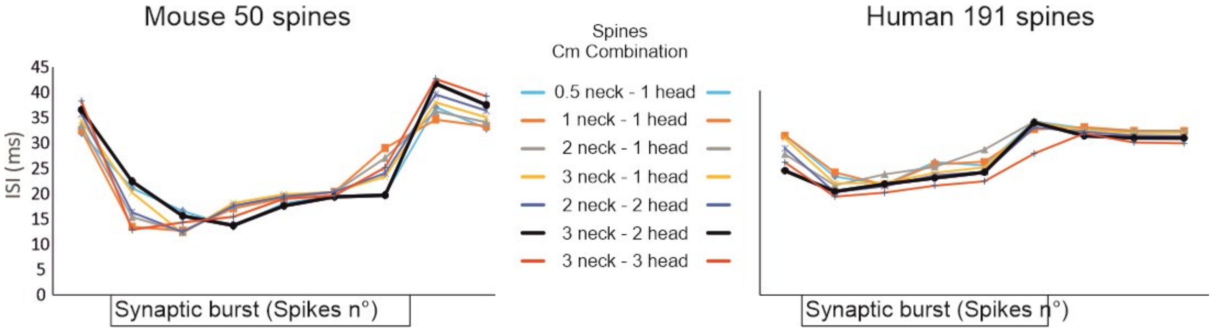

The consensus value for specific membrane capacitance, $C_m = 1\mu F/cm^2$, reflects the assumption that the composition of the neuronal membrane is identical throughout the neuron and across species. However, the precise C_m value may be influenced by several factors, including the density of ionic channels distributed on dendrites and spines heads or interneuron differences (e.g., PCs vs. pyramidal neurons). Given the limited availability of PC experimental data, a series of simulations was performed to evaluate the impact of different C_m values in the spine neck and head on PC electroresponsiveness. Different C_m combinations in spine neck and head were tested to evaluate the impact on synaptic inputs (50 synapses in mouse PCs and 191 synapses in human PCs chosen randomly in a ROI). Moving from low to high C_m values, the spikes generated by synaptic stimulation changed from 5 to 7 in mouse and from 4 to 5 in human PCs. At the same time, the spontaneous firing frequency changed from 32 to 26 Hz in mouse PCs and from 38 to 35 Hz in human PCs. These results showed a limited impact on the PC firing activity. The C_m combination that generated the sharpest burst-pause response, in line with available experimental data [6], was $3\mu F/cm^2$ in the spine neck and $2\mu F/cm^2$ in the head, both in mouse and human models.

Supplementary References

1. Yu, F. H., Yarov-Yarovoy, V., Gutman, G. A. & Catterall, W. a. Overview of molecular relationships in the voltage-gated ion channel superfamily. *Pharmacol. ...* **57**, 387–395 (2005).

2. Schneider, C. A., Rasband, W. S. & Eliceiri, K. W. NIH Image to ImageJ: 25 years of image analysis. *Nat. Methods* **9**, 671–5 (2012).

3. Masoli, S., Solinas, S. & D'Angelo, E. Action potential processing in a detailed Purkinje cell model reveals a critical role for axonal compartmentalization. *Front. Cell. Neurosci.* **9**, 1–22 (2015).

4. Masoli, S. & D'Angelo, E. Synaptic Activation of a Detailed Purkinje Cell Model Predicts Voltage-Dependent Control of Burst-Pause Responses in Active Dendrites. *Front. Cell. Neurosci.* **11**, 1–18 (2017).

5. Williams, S. R., Christensen, S. R., Stuart, G. J. & Häusser, M. Membrane potential bistability is controlled by the hyperpolarization-activated current/H in rat cerebellar Purkinje neurons in vitro. *J. Physiol.* **539**, 469–483 (2002).

6. Rapp, M., Segev, I. & Yarom, Y. Physiology, morphology and detailed passive models of guinea-pig cerebellar Purkinje cells. *J. Physiol.* **474**, 101–18 (1994).
