# Supplementary material for: Human Purkinje cells outperform mouse Purkinje cells in dendritic complexity and computational capacity
Source: Commun Biol. 2024 Jan 2;7:5. doi: 10.1038/s42003-023-05689-y (PMC10761885; doi:10.1038/s42003-023-05689-y)
Supplement: Supplementary file 4 — Reporting summary [file 42003_2023_5689_MOESM4_ESM.pdf]

nature portfolio

Corresponding author(s): Stefano Masoli PhD

Last updated by author(s): Nov 21, 2023

Reporting Summary

Nature Portfolio wishes to improve the reproducibility of the work that we publish. This form provides structure for consistency and transparency in reporting. For further information on Nature Portfolio policies, see our Editorial Policies and the Editorial Policy Checklist.

Statistics

For all statistical analyses, confirm that the following items are present in the figure legend, table legend, main text, or Methods section.

n/a	Confirmed	
☐	☒	The exact sample size (*n*) for each experimental group/condition, given as a discrete number and unit of measurement
☒	☐	A statement on whether measurements were taken from distinct samples or whether the same sample was measured repeatedly
☐	☒	The statistical test(s) used AND whether they are one- or two-sided<br>*Only common tests should be described solely by name; describe more complex techniques in the Methods section.*
☒	☐	A description of all covariates tested
☒	☐	A description of any assumptions or corrections, such as tests of normality and adjustment for multiple comparisons
☐	☒	A full description of the statistical parameters including central tendency (e.g. means) or other basic estimates (e.g. regression coefficient) AND variation (e.g. standard deviation) or associated estimates of uncertainty (e.g. confidence intervals)
☐	☒	For null hypothesis testing, the test statistic (e.g. *F*, *t*, *r*) with confidence intervals, effect sizes, degrees of freedom and *P* value noted<br>*Give P values as exact values whenever suitable.*
☒	☐	For Bayesian analysis, information on the choice of priors and Markov chain Monte Carlo settings
☒	☐	For hierarchical and complex designs, identification of the appropriate level for tests and full reporting of outcomes
☒	☐	Estimates of effect sizes (e.g. Cohen's *d*, Pearson's *r*), indicating how they were calculated

Our web collection on statistics for biologists contains articles on many of the points above.

Software and code

Policy information about availability of computer code

Data collection	Neurolucida 360. Clampex10.4, Multiclamp700B. NEURON 8. Python 3.
Data analysis	Neurolucida 360. Clampfit10, MS Excel, Matlab, OriginPro8, NEURON 8, BluePyOpt, Python 3.

For manuscripts utilizing custom algorithms or software that are central to the research but not yet described in published literature, software must be made available to editors and reviewers. We strongly encourage code deposition in a community repository (e.g. GitHub). See the Nature Portfolio guidelines for submitting code & software for further information.

Data

Policy information about availability of data

All manuscripts must include a data availability statement. This statement should provide the following information, where applicable:

- Accession codes, unique identifiers, or web links for publicly available datasets
- A description of any restrictions on data availability
- For clinical datasets or third party data, please ensure that the statement adheres to our policy

The most used models were uploaded on modeldb with a temporary access code which will be removed at publication time. The mouse morphologies are, a part of them, already available on neuromorpho.org. The human reconstructions will be made available on the same database. The models and experimental data will be uploaded on the eBRAINS platform as a requirement of the project.

Human research participants

Policy information about studies involving human research participants and Sex and Gender in Research.

Reporting on sex and gender	Not applicable.
Population characteristics	Not applicable.
Recruitment	Not applicable.
Ethics oversight	Not applicable.

Note that full information on the approval of the study protocol must also be provided in the manuscript.

Field-specific reporting

Please select the one below that is the best fit for your research. If you are not sure, read the appropriate sections before making your selection.

☒ Life sciences ☐ Behavioural & social sciences ☐ Ecological, evolutionary & environmental sciences

For a reference copy of the document with all sections, see nature.com/documents/nr-reporting-summary-flat.pdf

Life sciences study design

All studies must disclose on these points even when the disclosure is negative.

Sample size	The sample size was determined on the basis of literature and in order to guarantee a significance level.
Data exclusions	The morphological data was excluded in cases of bad filling or missing critical parts of the cell (i.e Dendrites). The electrophysiological data was excluded in case of a bad clamp, or non-conformity with the current knowledge about Purkinje cells behaviours in mice. In the case of Human recordings, the quality of the recordings was restricted by the number of biopsies. The computational models were discarded during the optimizations if they were too distant from the electrophysiological data. The validation process discarded all the models with: wrong I/O relationship, firing in the absence of a working AIS, wrong input resistance, and wrong synaptic responses.
Replication	All attempts at replication were successful.
Randomization	no randomization needed
Blinding	no blinding needed

Reporting for specific materials, systems and methods

We require information from authors about some types of materials, experimental systems and methods used in many studies. Here, indicate whether each material, system or method listed is relevant to your study. If you are not sure if a list item applies to your research, read the appropriate section before selecting a response.

Materials & experimental systems

n/a	Involved in the study	
☐	☒	Antibodies
☒	☐	Eukaryotic cell lines
☒	☐	Palaeontology and archaeology
☐	☒	Animals and other organisms
☒	☐	Clinical data
☒	☐	Dual use research of concern

Methods

n/a	Involved in the study	
☒	☐	ChIP-seq
☒	☐	Flow cytometry
☒	☐	MRI-based neuroimaging

Antibodies

Antibodies used	rabbit anti-LY antibody (1:400 000; generated at the Cajal Institute, CSIC, Madrid, Spain). biotinylated donkey anti-rabbit IgG (1:100; Amersham, Buckinghamshire, United Kingdom). Alexa fluor 488 streptavidin-conjugated (1:1000; Molecular Probes, Eugene, OR, United States of America)

Validation	WB

Animals and other research organisms

Policy information about studies involving animals; ARRIVE guidelines recommended for reporting animal research, and Sex and Gender in Research

Laboratory animals	C57 mouse with an average age of P27
Wild animals	The study did not involve wild animals.
Reporting on sex	No distinctions
Field-collected samples	The study did not involve samples collected from the field.
Ethics oversight	Ethical committee of the Italian Ministry of Health, Rome.

Note that full information on the approval of the study protocol must also be provided in the manuscript.
